# Therapeutic potential of Brazilian medicinal plants and their metabolites

**DOI:** 10.1590/1678-4685-GMB-2025-0222

**Published:** 2026-04-10

**Authors:** Juliana Mara Serpeloni, Fabio Vieira dos Santos, Katiuska Tuttis, Ilce Mara de Syllos Cólus

**Affiliations:** 1 Universidade Estadual de Londrina (UEL), Departamento de Biologia Geral, Laboratório de Mutagênese e Oncogenética (LAMON), Londrina, PR, Brazil.; 2 Universidade Federal de São João del Rei (UFSJ), Laboratório de Biologia Celular e Mutagênese (LaBCeM), Divinópolis, MG, Brazil.

**Keywords:** Biota/FAPESP, phytochemicals, natural products, chemotherapeutic drugs

## Abstract

Exploring natural products is essential for identifying new bioactive substances with unique molecular structures and mechanisms of action. Successful experiences in identifying and developing chemotherapeutic agents from plant-derived sources have inspired the search for new strategies and alternative treatments for various diseases. Today, more than half of the anticancer drugs in clinical use are derived, directly or indirectly, from natural products. In this review, we present findings from studies on medicinal plants conducted at the Laboratory of Mutagenesis and Oncogenetics, in collaboration with other research groups from the Program Biota/FAPESP, aimed at evaluating their safety for widespread use. Our research team has conducted extensive studies over approximately 25 years, assessing 65 crude plant extracts from 31 species and 8 isolated phytochemicals. Most of these species belong to the Melastomataceae family, followed by the Malpighiaceae family. Among the phytochemicals, flavonoids, diterpenes, indolinones, and alkaloids are the most common. In this comprehensive review, we examine the biological effects identified and their potential therapeutic applications, particularly in the context of anticancer strategies. Our goal is to highlight the pharmacological potential of compounds derived from these plants, thereby supporting the development of drugs that can be effectively utilized in clinical practice.

## Introduction

Brazil has the most incredible biodiversity of flora and fauna on the plane*t.* It is among the world’s 17 megadiverse countries, boasting two biodiversity hotspots: the Atlantic Forest and the Cerrado (Abranches, 2020). According to the Brazilian Ministry of Environment and Climate Change (Brazil, 2025), Brazil comprises 20% of the world’s catalogued animal and plant species, with at least 103,870 and 43,020 species, respectively. This biodiversity offers a wide variety of medicinal plants, and their use for therapeutic purposes has been a part of Brazilian culture since ancient times, originating from different traditional cultures, primarily those of Brazilian Indigenous peoples and Afro-descendants (Oliveira et al., 2021a). 

Approximately 82% of the Brazilian population utilizes herbal medicinal products in their healthcare, whether through traditional knowledge in communities, folk medicine practices transmitted orally from one generation to the next, or through official health systems (Ministério da Saúde, 2012). The Brazilian Ministry of Health (MS) implemented public policies in 2006, following World Health Organization (WHO) resolutions, to expand access to therapeutic alternatives, promote the sustainable use of biodiversity, and develop and integrate these alternatives into health services (Brazil, 2006).

Although many people consider natural products safer and more beneficial than industrialized compounds due to their lower cost and fewer side effects, plants actively produce a wide array of secondary metabolites to defend themselves against biotic and abiotic stressors. These phytochemicals, including carotenoids, flavonoids, alkaloids, polyphenols, nitrogenous and organosulfur compounds, can exhibit cytotoxic and genotoxic properties, posing potential risks to human health (Akhtar et al., 2020). The chemical diversity and ethnopharmacological relevance of plant-derived compounds remain valuable for drug discovery and development. In Brazil, ANVISA mandates pharmacological, toxicogenomic, and efficacy-safety assessments for all new herbal medicines derived solely from plant materials (Agência Nacional de Vigilância Sanitária, 2014).

Natural products have long served as sources of anticancer agents, with several FDA-approved drugs, such as paclitaxel, vincristine, vinblastine, and etoposide, originating from plant-derived compounds. An extensive scientific review demonstrated that, among all small-molecule medicines approved between 1981 and 2019, approximately 50% are natural products, their derivatives, or synthetic compounds containing pharmacophoric groups inspired by natural product structures. For anticancer agents, this proportion increases to about 65% (Newman and Cragg, 2020). Given the high systemic toxicity of conventional anticancer drugs, which often affect both tumor and normal cells, there is a growing interest in identifying new compounds with selective cytotoxicity toward cancer cells and minimal harm to healthy tissues.

In Brazil, research on natural products gained momentum with the launch of the BIOTA-FAPESP Program in 1999 by FAPESP, which aimed to characterize, conserve, restore, and sustainably use biodiversity. This initiative later evolved into the Biota Network for Bioprospection and Bioassays (BIOprospecTA), integrating research groups nationwide. Within this framework, a team of researchers from São Paulo, coordinated by Prof. Dr. Wagner Vilegas, invited LAMON to participate and conduct *in vitro* and *in vivo* assessments of extracts from medicinal plants traditionally used to treat gastrointestinal disorders. Later, continuing the initial project, we developed the project: “Standardised extracts for the treatment of chronic diseases,” with the objectives of (i) deepening pharmacological tests and searching for new plants that could be included in the RENISUS (from Portuguese, Relação Nacional de Plantas Medicinais de Interesse ao Sistema Único de Saúde) list, and (ii) standardizing the most promising plant extracts from a chemical, qualitative, and quantitative point of view. These interdisciplinary efforts enabled the isolation and characterization of key phytochemicals from selected plant extracts, as well as the evaluation of their biological activities *in vitro* and *in vivo*. 

This review focuses on several vegetal extracts and some active compounds derived from *Croton cajucara Benth (C. cajucara)*, *Fridericia platyphylla* (Cham.) L.G. Lohmann (*F. platyphylla,* syn. *Arrabidaea brachypoda* Bureau), *Indigofera suffruticosa* Miller (*I. suffruticosa), and I. truxillensis* Kunth (*I. truxillensis*), whose extracts have shown promising results.

## Materials and Methods

### The process of study selection and extracting information

This review presents an overview of the research on phytochemicals and plant extracts from Brazilian flora conducted at LAMON. It encompassed various types of investigations done in our laboratory over approximately 25 years of study, including (a) studies that examined the biological effects of various plant extracts and phytochemicals on animal models (*in vivo*), and in a controlled laboratory environment (*in vitro*) and (b) investigations that explored the underlying mechanisms of action of these natural products.

At the beginning of this review, LAMON’s publications were searched to separate those referring to vegetal extracts and isolated phytochemicals. The articles examined underwent a comprehensive evaluation process that considered various factors, including the year of publication, the specific substance being studied, the experimental framework employed, the concentrations evaluated, the outcomes observed, and the main results. The searches revealed 40 studies that met these criteria. Among the species evaluated, six were assessed before the Biota project: *Croton cajucara* Benth., four *Miconia* species (*M. cabucu* Hoehne*, M. rubiginosa* (Bonpl.) DC.*, M. stenostachya* DC.*,* and *M. albicans* (Sw.) Steud.), and *Anacardium occidentale* Linn.

### Ethical issues

All experiments performed on animals were previously submitted to the Animal Research Ethics Committee, as reported in each cited article.

## Results

### Plant species studied

In total, 32 vegetable species were evaluated at LAMON over 25 years. In *Scoparia dulcis* Linn., only the active compound cirsimarin (CIR) was assessed. 

An ethnopharmacological inventory conducted in the Cerrado biome, covering the states of São Paulo, Tocantins, and Mato Grosso, served as the starting point for our project. This involved the chemical analysis of extracts and infusions from medicinal plants commonly used as natural remedies by traditional communities in these regions. Most species studied belonged to the Melastomataceae family (6), followed by Malpighiaceae (5), and Euphorbiaceae and Fabaceae (4). These angiosperm families are among the most prevalent in Brazil, with Fabaceae and Malpighiaceae particularly dominant in the Cerrado savanna (Alvarez et al., 2025; The Brazil Flora Group, 2022).

All plant species evaluated are summarized in Table 1, along with their common names, families, traditional uses, and main active ingredients.

Among the most common traditional uses are the prevention of gastrointestinal disorders (such as diarrhea, ulcers, nausea, and vomiting), as well as anti-inflammatory and antimicrobial applications. The phytochemical profile varies significantly between species and depends on the extraction method used, including methanolic or ethanolic extracts and their respective fractions.

**Table 1:** Main vegetal species evaluated in the Laboratory of Mutagenesis and Oncogenetics, their popular name, family, traditional use, and main active ingredients.

Species	Popular Name	Family	Main traditional use	Main active ingredients	Ref.
*Alchornea castaneifolia* (Humb. & Bonpl. ex Willd. A.Juss.	Sarã, sarão, gurupiá	Euphorbiaceae	Rheumatism, arthritis, pain-relieving, diarrhea, cold, aphrodisiac, tonic for the reproductive system	**1. EFF obtained from HEL:** Flavonoid derivatives (quercetin-3-*O*-β-d-galactopyranoside; quercetin-3-*O*-α-l-arabinopyranoside; myricetin-3-*O-*α-l-arabinopyranoside; quercetin, and amentoflavone; gallic acid derivatives (gallic acid and gallic acid methyl ester	Pott A and Pott VJ, 1994; Duke and Vasquez, 1994; Hiruma-Lima et al., 2006
*Alchornea glandulosa* Poepp.	Tapiá, maria-mole, iricurana, boieiro, araribá, bugé, tamanqueiro, tapiá-guaçu, tapiá-mirim, caixeta, canela-raposa	Euphorbiaceae	Rheumatism, arthritis, colds, muscle pain	**1. MeOH:** Flavonoid derivatives (myricetin-3-*O*-α-l-rhamnopyranoside; quercetin-3-*O*-β-d-galactopyranoside; quercetin-3-*O*-α-l-arabinopyranoside; quercetin, and amentoflavone; gallic acid derivatives (gallic acid and methyl gallate	Lorenzi, 1998; Calvo et al., 2007
*Alchornea triplinervia* (Spreng. Müll.Arg.	Tanheiro, tapiá, tanaeiro, tapiá-guaçu, lapiá-guaçu-branco, tapiá-vermelho,caixeta, pau-jangada, tapiaeiro, boleira, pau-de-tamanco, pau-do-tanho	Euphorbiaceae	Gastric disorders	**1. MeOH:** Quercetin, quercetin-7-*O*-β-d-glucopyranoside; quercetin-3-*O*-β-d-glucopyranoside; quercetin-3-*O*-β-d-galactopyranoside; quercetin-3-*O*-α-l-arabinopyranoside; amentoflavone; brevifolin carboxylic acid; gallic acid; methylgallate; 2. CHCl₃: stigmasterol; campesterol; sitosterol; lupeol; friedelan-3-ol; friedelan-3-one	Lorenzi, 1998; Calvo et al., 2010; Bonacorsi et al., 2013
*Anacardium occidentale* L.	Cajueiro, acajaíba, acaju, acajuíba, caju-manso, caju-banana, caju-manteiga	Anacardiaceae	Diabetes, inflammation, ulcers, aphthous ulcers, antiseptic, vermifuge, intestinal colic, cough	**1. EB:** Gallic acid; Gallic acid-galloyl; B-type rocyanidin Dimer; Epicatechin gallate; Hexose polymer 2. EL: Monogalloyl-diglucose; Gallic acid; Gallic acid-galloyl Hexoside; Tetra-Ogalloylhexoside; Quercetin *O*-hexoside; Pentagalloyl-hexoside Isomer; Hexagalloyl hexoside; Luteolin; Quercetin galloyl-*O*-eoxy-hexoside; Agathisflavone 3. CAJ: Ascorbic acid; anacardic acids; quercetin; tannins; phenols; carotenoids	Corrêa, 1984; Lorenzi, 1998; Barcelos et al., 2007b; Barcelos *et al.,* 2007a; Costa et al., 2020
*Bauhinia holophylla* (Bong. Steud.	Unha-de-vaca, pata-de-vaca-do-cerrado	Fabaceae	Diabetes, diuretics, obesity	**1. HEL:** Quercetin-o-pentoside; quercetin-o-deoxyhexoside; quercetin-o-hexoside	Rodrigues and Carvalho, 2001; Durigan et al., 2018; Ribeiro et al., 2018
*Byrsonima basiloba* A. Juss	Murici, murici-do-campo	Malpighiaceae	Diarrhea, gastric ulcer	**1. MeOH:** (+-catechin; quercetin-3-*O*-l-arabinopyranoside; quercetin; gallic acid; methyl gallate; amentoflavone 2. CHCl₃: Lupeol; ursolic acid; oleanolic acid	Lorenzi, 1998; Lira et al., 2008
*Byrsonima correifolia* A. Juss.	Murici-pitanga	Malpighiaceae	Antiulcerogenic, antimicrobial	**1. MeOH/H₂O:** Proanthocyanidins; amentoflavone derivatives; quercetin derivatives; galloylquinic acid derivatives	Sousa et al., 2015; Specian et al., 2016; Specian et al., 2023
*Byrsonima crassifolia* (L. Kunth (syn. *Byrsonima fagifolia* Nied.	Murici-do-campo	Malpighiaceae	Gastroprotective, antidiarrheal	**1. Infusion:** Monogalloylquinic acids; digalloylquinic acids; trigalloylquinic acid; tetragalloylquinic acids; pentagalloylquinicacids and ﬂavonoid glycosides	Corrêa, 1978a; Sannomiya et al., 2007b
*Byrsonima intermedia* A. Juss.	Cangica, murecy, mirici	Malpighiaceae	Gastroprotective, antidiarrheal, diuretic, antiasthmatic	**1. MeOH:** Quercetin-3-*O*-β-d-galactopyranoside; (+-catechin; (−-epicatechin; gallic acid; methyl gallate; quercetin-3-*O*-α-l-arabinopyranoside; amentoflavone; quercetin-3-*O*-(2″-*O*-galloyl-β-galactopyranoside; quercetin-3-*O*-(2″-*O*-galloyl-α-arabinopyranoside; quercetin	Corrêa, 1926; Sannomiya et al., 2007b; Specian et al., 2016; Specian et al., 2023
*Byrsonima verbascifolia* Rich. ex Juss.	Douradinha falsa, murici-assú, murecy	Malpighiaceae	Inhibit the replication of rotavirus, inflammation, diarrhea	**1. Different extracts:** Galloyl quinic acid derivatives; amentoflavone; catechins; quercetin derivatives	Corrêa, 1926; Panizza, 1997; Specian et al., 2016; Specian et al., 2023
*Croton cajucara* Benth.	Sacaca, Cajucara	Euphorbiaceae	Diabetes, diarrhea, gastrointestinal disorders, liver diseases	**1. MeOH:** Terpenoids	Maciel et al., 2000; Santos et al., 2006
*Davilla elliptica* A.St.-Hil.	Lixeirinha, lixeira-rasteira, Iixinha	Dilleniaceae	Stomach diseases, diarrhea, swelling	**1. MeOH:** Myricetin and quercetin derivatives; gallic acid derivatives	Pott A and Pott VJ, 1994; Biso et al., 2010
*Davilla nitida* (Vahl Kubitzki	Lixeirinha	Dilleniaceae	Gastric problems	**1. MeOH:** Myricetin and quercetin derivatives; gallic acid, tannins	Biso et al., 2010; Bieski et al., 2012
*Fridericia platyphylla* (Cham. L.G.Lohmann (syn. *Arrabidaea brachypoda* Bureau	Cipó una	Bignoniaceae	Kidney stones, joint pain	**1. EtOH/H₂O:** brachydin A; brachydin B; brachydin C; brachydin D; brachydin E; brachydin F; brachydin G; brachydin H; brachydin I; brachydin J	Corrêa, 1926; Rocha et al., 2015; Serpeloni et al., 2020
*Indigofera suffruticosa* Mill.	Anileira verdadeira	Fabaceae	Antimicrobial, anti-spasmodic, sedative, diuretic, anti-epileptic, stomach and urinary diseases, fevers, hepatitis	**1. MeOH:** kaempferol and quercetin derivatives; indigo; indirubin	Calvo et al., 2011; Corrêa, 1926
*Indigofera truxillensis* Kunth	Anileira	Fabaceae	antiulcerogenic and antioxidant	**1. MeOH:** kaempferol and quercetin derivatives; indigo; indirubin	Calvo et al., 2011; Corrêa, 1926
*Machaerium hirtum* (Vell. Stellfeld	Barreiro, barreirinho, espinheiro	Fabaceae	Diarrhea, cough, cancer	**1: EtOH:** saponarin; isovitexin; isoorientin; swertisin apigenin-6-C-β-d-glucopyranosyl-8-C-β-d-xylopyranoside; apigenin-8-C-β-d-glucopyranosyl-7-*O*-β-d-glucopiranoside	Pott A and Pott VJ, 1994; Ignoato et al., 2013; Ribeiro et al., 2016
*Miconia albicans* (Sw. Steud.	Olhos-de-porco, canela-de-velho	Melastomataceae	Fever, vitiligo	**1. MeOH:** Quercetin; quercetin-3-O-glucoside; rutin; 3-(E-p-coumaroyl-α-amyrin 2. CHCl_3:_ α-amyrin; epi-betulinic acid; ursolic acid; epi-ursolic acid	Corrêa, 1978b; Albuquerque et al., 2007
*Miconia formosa* Cogn. (syn*. Miconia cabucu* Hoehne	Pixirica, pixiricão	Melastomataceae	Stomach pain	**1. MeOH:** flavonoids; tannins; phenolic compounds; biflavonoid 5-hydroxy-4′; 7-dimethoxyflavone-(6-C-6″-5″-hydroxy-3″′,4″′,7″-trimethoxyflavone, gallic acid; quercetin-3-o-β-xylopyranosyl-(1→2-o-α-rhamnopyranoside; quercetin-3-o-α-rhamnopyranoside; myricetin-3-o-α-rhamnopyranoside; quercetin-3-o-β-glucopyranoside; kaempferol-3-o-β-(6′’-coumaroyl-glucopyranoside; myricetin-3-o-β-xylopyranosyl-(1→2-o-α-rhamnopyranoside	Lorenzi, 1998; Bourdy et al., 2004; Rodrigues et al., 2007
*Miconia rubiginosa* (Bonpl. DC.	Capiroroquinha	Melastomataceae	Throat infections, colds, fever	**1. MeOH:** gallic acid; quercetin-3-o-α-rhamnopyranoside; quercetin-3-o-β-arabinofuranoside; quercetin-3-o-α-arabinopyranoside; quercetin-3-o-β-arabinopyranoside; quercetin-3-o-β-galactopyranoside; quercetin-3-o-α-rhamnopyranosil-(1→4-o-β-galactopyranoside; epicatechin	Rodrigues and Carvalho, 2001; Serpeloni et al., 2008a
*Miconia stenostachya* DC*.*	Guamirim-do-cerrado, papa-terra, canela-de-velha	Melastomataceae	Throat infections	**1. MeOH:** glycosylated flavonoids derived from quercetin and myricetin; catechin derivatives	Durigan et al., 2018; Serpeloni et al., 2011
*Mouriri elliptica* Mart.	Coroa-de-frade	Melastomataceae	Gastric ulcer	**1: MeOH:** myricetin-3-*O*-β-galactopyranoside; myricetin-3-*O*-β-glycopyranoside; myricetin-3-*O*-β-rhamnopyranoside 4; quercetin-3-*O*-β-galactopyranoside; quercetin-3-*O*-β-xylopyranoside; quercetin-3-*O*-α-rhamnopyranoside	Pott A and Pott VJ, 1994; Santos et al., 2008a
*Mouriri pusa* Gardner	Puçá, mandapuçá, manapuçá, puçá-preta, jabuticaba-do-campo, jabuticaba-do-cerrado, munduru, moroso-cigano	Melastomataceae	Gastric ulcer	**1. MeOH:** myricetin-3-*O*-β-galactopyranoside; myricetin-3-*O*-β-glycopyranoside; rutin; myricetin-3-*O*-β-rhamnopyranoside 4; quercetin-3-*O*-β-galactopyranoside; quercetin-3-*O*-β-xylopyranoside; quercetin-3-*O*-β-arabinopyranoside; quercetin-3-*O*-α-arabinofuranoside; kaempferol-3-3-*O*-β-galactopyranoside; quercetin-3-*O*-α-rhamnopyranoside; quercetin; myricetin	Lorenzi, 1998; Santos et al., 2008b
*Myrcia bella* Cambess.	Mercurinho	Myrtaceae	Diabetes, hemorrhages, hypertension	**1. EtOH/H₂O:** Eleven flavonoid-*O*-glycosides; six acylated flavonoid derivatives of myricetin and quercetin; caffeic acid; ethyl galate; gallic acid; quinic acid	Pott et al., 2006; Saldanha et al., 2013; Serpeloni et al., 2015; Marena et al., 2022
*Pouteria ramiflora* (Mart. Radlk.	Leiteiro-preto, abiu, abiu-carriola, massaranduba, ibacoixa, guajara, mandapuca, grão-de-galo, pitomba-de-leite	Sapotaceae	Dysentery, pain, inflammation, hyperlipidemia, ulcers, kidney disorders, antinociceptive, anti-inflammatory	**1. EtOH/H₂O:** Gallic acid; Quinic acid; Apigenin-C-hexosyl; Quercetin-*O*-pentoside; Quercetin-*O*-deoxyhexoside; Myricetin-*O*-pentoside; Quercetin-*O*-hexoside; Myricetin-*O*-deoxyhexoside; Myricetin-*O*-hexoside; Apigenin-C-pentosyl-C-deoxyhexoside; Apigenin-C-hexosyl-C-pentoside; Apigenin-C-hexosyl-C-deoxyhexoside; Apigenin-di-C-hexosyl	Lorenzi, 1992; Tuttis et al., 2021
*Qualea grandiflora* Mart.	Pau-terra-do-campo, pau-terra-do-cerrado, ariauá	Vochysiaceae	Diarrhea, nausea, vomiting, back pain	**1. MeOH:** 28-nor-17, 22-seco-2α, 3β, 19, 22, 23-pentahydroxy-Δ12-oleanane; Bellericagenin B; Arjunglucoside I; 3, 3′-di-*O*-methylellagic acid-4-*O*-β-d-glucopyranoside; 3-*O*-methylellagic acid-4′-*O*-α-l-rhamnopyranoside; 3, 3′,4-tri-*O*-methylellagic acid-4′-*O*-β-d-glucopyranoside; 3, 3′-di-*O*-methylellagic acid 2. CHCl_3_: β-Sitosterol glucoside; Friedelin; Lupenone 3. AqE: 28-nor-17, 22-seco-2α, 3β, 19, 22, 23-pentahydroxy-Δ12-oleanane; Bellericagenin B; 3, 3′-di-*O*-methylellagic acid-4-*O*-β-d-glucopyranoside; 3-*O*-methylellagic acid-4′-*O*-α-l-rhamnopyranoside; 3, 3′,4-tri-*O*-methylellagic acid-4′-*O*-β-d-glucopyranoside	Corrêa, 1978b; Santos et al., 2011
*Qualea multiflora* Mart.	Cinzeiro, pau de tucano, pau terra do campo, uva-puva do campo	Vochysiaceae	External ulcers, gastric disorders, inflammation	**1. MeOH:** 8-nor-17, 22-seco-2α, 3β, 19, 22, 23-pentahydroxy-Δ12-oleanane; Bellericagenin B; Arjunglucoside I; Bellericaside B; 3, 3′-di-*O*-methylellagic acid-4-*O*-β-d-glucopyranoside; 3-*O*-methylellagic acid-4′-*O*-α-l-rhamnopyranoside; 3, 3, 3′-di-*O*-methylellagic acid 2. CHCl_3_: β-Sitosterol glucoside; Friedelin; Lupenone; β-Sitosterol 3. H_2_O: 28-nor-17, 22-seco-2α, 3β, 19, 22, 23-pentahydroxy-Δ12-oleanane; Bellericagenin B; Arjunglucoside I; Bellericaside B; 3, 3′-di-*O*-methylellagic acid-4-*O*-β-d-glucopyranoside; 3, 3′,4-tri-*O*-methylellagic acid-4′-*O*-β-d-glucopyranoside	Corrêa, 1926; Santos et al., 2011
*Qualea parviflora* Mart.	Pau-terra-de-flor-miudinha, pau-terra-mirim, pau-terra, coatá-quiçaua	Vochysiaceae	Gastric disorders, antiseptic, anti-inflammatory, astringent	**1. MeOH:** 3,3′-di-*O*-methylellagic acid-4-*O*-β-d-glucopyranoside; 3-*O*-methylellagic acid-4′-*O*-α-l-rhamnopyranoside; 3,3′-4-tri-*O*-methylellagic acid-4′-*O*-β-d-glucopyranoside; 3,3′-di-*O*-methylellagic acid; triterpenes; saponins	Corrêa, 1978b; Mazzolin et al., 2010
*Serjania marginata* Casar.	Timbó, cipó-timbó	Sapindaceae	Stomach pain	**1. EtOH/H₂O:** 3-*O*-d-β-glucopyranosylsitosterol; pulsatilla saponin D; hederacolchiside A1; salzmannianoside B; 3-*O*-α-l-arabinopyranosyl(1→3-α-l-rhamnopyranosyl(1→2[β-d-glucopyranosyl(1→4]-α-l-arabinopyranosyloleanolic acid; quercetin 3-*O*-α-l-rhamnopyranoside; epicatechin; cassiaoccidentalin A; tetrastigma B; apigenin 6-C-β-boivinopyranosyl-7-*O*-β-d-glucopyranoside; apigenin 6-C-[2-*O*-α-l-rhamnopyranosyl(1→2]-β-d-xylopyranoside; apigenin 7,5″-anhydro-8-C-α-(2,6-dideoxy-5-hydroxy-ribo-hexopyranosyl-4′-*O*-β-d-glucopyranoside; proanthocyanidins A-1 and A-2; cinnamtannin B-1	Corrêa, 1978b; Bourdy et al., 2004; Heredia-Vieira et al., 2015
*Strychnos pseudoquina* A.St.-Hil.	Quina, quina-do-cerrado, quina-grossa	Loganiaceae	Liver and stomach diseases, fever, malaria	**1. MeOH:** rutin; kaempferol 3-*O*-b-rutinoside; nordiidrofluorocurarine; flavonoids; terpenoids; waxes	Pott A and Pott VJ, 1994; Silva et al., 2005a

CAJ: Cashew Apple Juice; CHCl₃: Chloroform Extract; EB: Ethanolic Bark Extract; EFF: Enriched Flavonoidic Fraction; EL: Ethanolic Leaf Extract; AqE: Aqueous extract; EtOH/H₂O: Hydroethanolic extract; HEL: Hydroethanolic Extract of the Leaves; MeOH: Methanolic Extract; MeOH/H₂O: Hydromethanolic extract

### Genotoxicity and mutagenicity parameters

Evaluation of genotoxicity and mutagenicity plays a critical role in managing chemical risks to human health and environmental balance, primarily because genotoxic events can lead to irreversible and severe biological consequences. 

The term genotoxicity refers to the capacity of agents to damage the genetic material, either directly or indirectly, leading to alterations such as chromosomal strand breaks, cross-links, or oxidative lesions on DNA nucleotides. On the other hand, mutagenicity refers to the induction of permanent and transmissible changes in the DNA sequence (e.g., point mutations, insertions, or deletions), which can result in altered gene function or chromosomal abnormalities. In this way, not all genotoxic agents are mutagenic, as some may cause DNA damage that is either repaired or leads to non-mutagenic outcomes such as cell death.

According to the International Conference of Harmonization (ICH), S2 (R1) on genotoxicity testing and data interpretation for pharmaceuticals intended for human use guide (Food and Drug Administration, 2020), genotoxicity studies include both *in vitro* and *in vivo* tests designed to detect the potential of the substances under investigation to cause gene mutations and chromosomal alterations. This guideline recommends one of two test batteries. The first battery consists of i) a gene mutation test in bacteria; ii) a cytogenetic test to assess chromosomal damage (*in vitro* chromosomal aberration or micronucleus test) or an *in vitro* gene mutation test in mouse lymphoma TK cells; and iii) an *in vivo* genotoxicity test (micronuclei or chromosomal aberrations). The second battery recommends i) a gene mutation test in bacteria and ii) an *in vivo* genotoxicity assessment in two tissues, usually a micronucleus test in rodent hematopoietic cells and a second *in vivo* assay.

The battery of assays recommended by regulatory agencies is based on the specific application of each test to detect different types of genetic damage. A fundamental understanding of their underlying principles is essential for accurately interpreting the results obtained.

The *Salmonella*/mammalian microsome mutagenicity test, widely known as the Ames test, is a well-established method for identifying substances that induce gene mutations, either directly or indirectly (Zeiger, 2019). The test employs genetically engineered *Salmonella enterica* sorovar Typhimurium and *Escherichia coli* strains, each carrying distinct mutations in genes essential for amino acid biosynthesis (histidine in *Salmonella* and tryptophan in *E. coli*). These mutations prevent bacterial growth in the absence of the required amino acid, unless a reversion event restores prototrophy. The strains vary in their DNA repair capacity, mutation targets, and spontaneous reversion frequencies. Compounds must be tested both with and without a metabolic activation system (S9 mix) to evaluate the mutagenic potential of both the test compound and its metabolic derivatives (Zeiger, 2023).

Besides gene mutations, another key endpoint to be evaluated is the induction of chromosomal damage, which can affect both chromosome structure and number. Such damage may arise through two distinct mechanisms: clastogenesis, resulting from double-strand DNA breaks and leading to chromosomal fragmentation, or aneugenesis, caused by errors in chromosome segregation, leading to numerical alterations. The classical method for detecting these events at the cytogenetic level is the chromosomal aberration (CA) test, which involves labor-intensive microscopic examination of metaphase cells to count chromosomes and identify different types of structural alterations (Guimarães et al., 2014).

In contrast, the micronucleus (MN) assay provides a simple and efficient method for detecting both clastogenic and aneugenic events (Fenech, 2000) by identifying micronuclei -small membrane-bound chromatin bodies formed when acentric fragments or entire chromosomes are excluded from daughter nuclei during cell division (Witt et al., 2025). The assay can be performed *in vitro*, most commonly using the cytokinesis-block micronucleus (CBMN) protocol (Organization for Economic Co-Operation and Development, 2023), or *in vivo*, typically in rodent models (Organization for Economic Co-Operation and Development, 2016).

The single-cell gel electrophoresis (SCGE) assay, commonly known as the comet assay, is a sensitive technique that complements information from both the Ames and micronucleus tests by detecting primary DNA damage induced by genotoxic agents in eukaryotic cells. The assay relies on the relaxation of negatively supercoiled DNA loops in agarose-embedded nucleoids after cell lysis. DNA strand breaks facilitate the migration of relaxed loops toward the anode during electrophoresis, resulting in a comet-like structure in which the tail intensity correlates with the extent of DNA damage. In addition to detecting strand breaks, the comet assay incorporates lesion-specific DNA repair enzymes to identify oxidized and alkylated bases, and it also measures DNA cross-links, bulky adducts, methylation status, and DNA repair kinetics (Azqueta and Collins, 2013; Collia et al., 2024). Its applicability spans *in vitro* and *in vivo* genotoxicity testing, human biomonitoring, ecogenotoxicology, and mechanistic studies on DNA damage and repair. 

**Figure 1:**
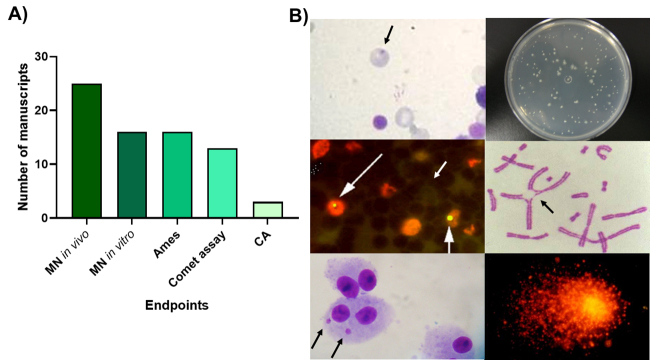
a) Number of manuscripts published with each genotoxicity/mutagenicity test in the Mutagenesis and Oncogenetics Laboratory. b) Representative images of the micronucleus assays *in vivo* (micronucleated polychromatic erythrocyte (PCE) from bone marrow and micronucleated peripheral blood reticulocytes, MNRET), *in vitro* (ACP02 cells), Ames test (*Salmonella* Typhimurium TA98), chromosome aberration (CA) (CHO-K1 cells), and comet assay (peripheral blood).

The publications conducted at LAMON with crude extracts and phytochemicals focused on using these recommended tests, mainly the MN test *in vivo* (25), *in vitro* (16), the Ames test (16), and the comet assay (13). The chromosome aberration assay was employed in only 3 studies (Figure 1A). Representative images of these tests, obtained in our experiments, are shown in Figure 1B.

Previous studies by LAMON focused on assessing the genotoxic and mutagenic profiles of extracts and active compounds. More recent *in vitro* research has expanded to explore additional parameters to elucidate their mechanisms of action. These parameters will be briefly presented in the discussion section.

### Vegetal extracts and their biological activities

Tables 2 and 3 show the biological activities of the main vegetal extracts evaluated by LAMON. In total, 37 studies were conducted *in vitro and 22 in vivo.*

**Figure 2:**
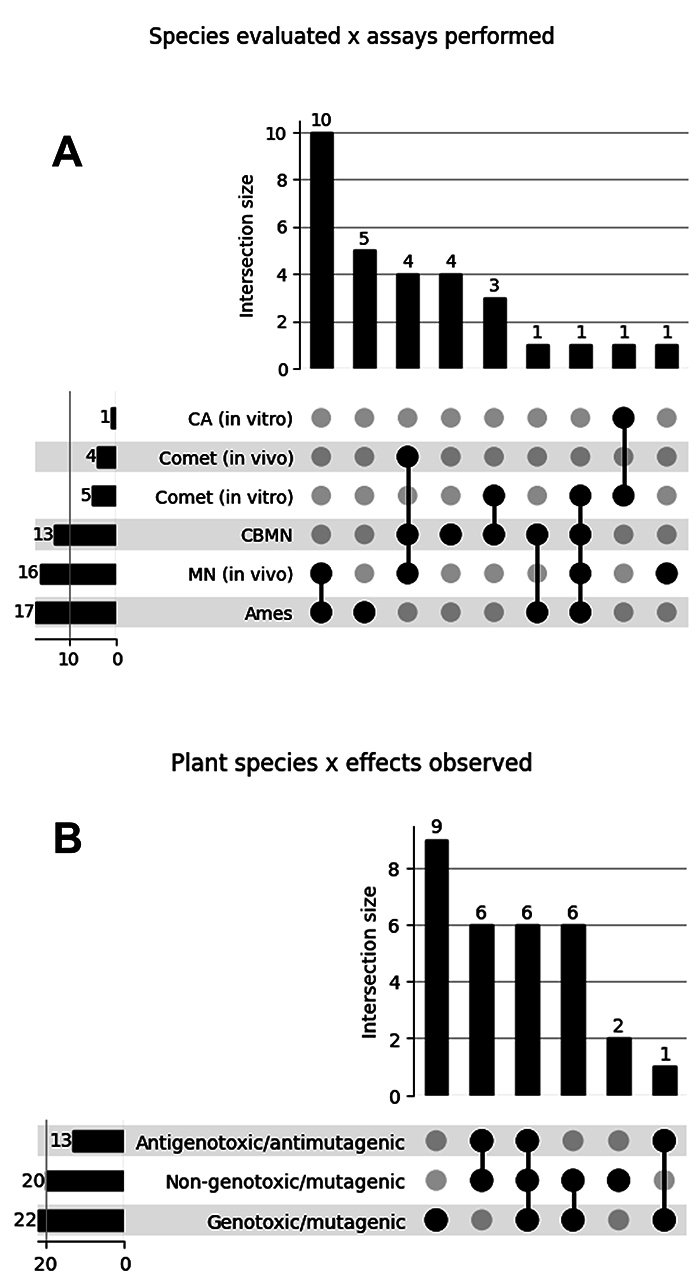
Intersection plots summarizing the evaluation of plant species. (A) Assays applied to each species, with bars representing the number of species tested in each assay combination; (B) Biological outcomes reported, showing how species can simultaneously present different profiles, depending on the derived compound evaluated or assay employed.

Among the 32 species investigated, 30 had at least one plant extract evaluated for genotoxic, mutagenic, and/or chemopreventive effects using a variety of *in vitro* and *in vivo* assays. Figure 2 summarizes these data, illustrating in panel (A) the number of species assessed across different assay combinations. For instance, 17 species had at least one extract tested with the Ames assay, while 10 species were simultaneously evaluated using both the Ames test and the *in vivo* micronucleus assay. Overall, researchers investigated the majority of these species in at least two distinct experimental models.

**Table 2:** Main vegetal extracts evaluated in the Laboratory of Mutagenesis and Oncogenetics and their biological activities assessed *in vitro.*

Species/organ	Assay	Model	Parameter	Extract	Concentrations (Number/range) Ames (mg/plate) Cell culture (µg/mL)		Ref.
*Alchornea castaneifolia* Leaves	Ames Assay	*S.* Typhimurium *(TA98, TA100, TA97a, TA102)*	Gene mutation	a. MeOH b. CHCl₃	a. 5 (3.63 - 29.04) b. 5 (1.01 - 8.1)	a: mutagenic: TA98 (-S9/+S9) > 14.52 b: non-mutagenic	Santos et al., 2010
*Alchornea glandulosa* Leaves	Ames Assay	*S*. Typhimurium *(TA98, TA100, TA97a, TA102)*	Gene mutation	a. MeOH b. CHCl₃	a. 5 (2.83- 22.7) b. 5 (1.74 -13.9)	a: mutagenic: TA98 (-S9/+S9), TA97a (-S9) > 5.67 b: non-mutagenic	Santos et al., 2010
*Alchornea triplinervia* Leaves	Ames Assay	*S*. Typhimurium *(TA98, TA100, TA97a, TA102)*	Gene mutation	a. MeOH b. CHCl₃	a. 5 (3.1 - 25) b. 5 (2.1 - 16.6)	a: mutagenic: TA98 (+S9), TA97a (+S9) all concentrations b: non-mutagenic	Calvo et al., 2010
*Anacardium occidentale* Bark	CA analysis	V79 cell line	Mutagenicity Antimutagenicity	MeOH	- 3 (500 - 2000)	- non-mutagenic - antimutagenic: all concentrations + DXR (SIM)	Barcelos et al., 2007b
*Anacardium occidentale* Bark	Clonogenic Assay Comet Assay	V79 cell line	Cell Survival Genotoxicity Antigenotoxicity	MeOH	- 7 (500 - 6000): Cell Survival - 3 (500 - 2000): Genotoxicity, Antigenotoxicity	- cytotoxicity > 3000 - genotoxicity: 2000 - antigenotoxicity: 1000 + MMS (PRE) - antigenotoxicity: all concentrations + MMS (SIM, POST)	Barcelos et al., 2007a
*Bauhinia holophylla* Leaves	MTT Apoptosis/Necrosis CBMN Flow cytometry RT-qPCR	HepG2 cell line	Cytotoxicity Mutagenicity Antimutagenicity Cell cycle analysis Gene expression	EtOH/H₂O	- 10 (5 -50): MTT - 3 (7.5 - 30): CBMN, Apoptosis, Flow Cytometry - 1 (7.5): RT-qPCR	- ↓ cell viability > 10 (72h) - apoptotic effects 30 (24h) - antiapoptotic effects: 7.5 (PRE, SIM, POST) - non-mutagenic - ↓ proliferation: all concentrations (< NDI) - antimutagenic: 7.5 + B[a]P) (PRE, SIM, POST) - cell cycle: ↓ G1 and ↑ S (all concentrations) - ↑ S (7.5 + B[a]P) (SIM, POST); ↑ G1, ↓ G2/M (PRE) - ↑ *CYP1A1* (7.5); ↑ *CYP1A1*, *XPC* (7.5 + B[a]P) (POST)	Ribeiro et al., 2018
*Byrsonima basiloba* Leaves	Ames Assay	*S*. Typhimurium *(TA98, TA100, TA97a, TA102)*	Gene mutation Antimutagenicity	a. MeOH b. CHCl₃	a. 5 (3.65 - 29.2): Mutagenicity a. 10 (0.46 - 21.9): Antimutagenicity b. 5 (1.99 - 15.9): Mutagenicity b. 5 (0.99 - 15.9): Antimutagenicity	a,b: non-mutagenic a: antimutagenic (0.46 - 1.83) + AFL) (SIM - TA98, TA100) a: antimutagenic (0.46 - 7.3) + B[a]P) (SIM - TA98) a: antimutagenic ((1.82 - 7.3) + H₂O₂) (SIM - TA102) a: antimutagenic (> 7.3 + MMC) (SIM - TA102) a: antimutagenic (> 1.82 + NPD) (SIM - TA98) b: antimutagenic (all concentrations + NPD) (SIM - TA98,TA97a) b: antimutagenic (all concentrations + B[a]P (SIM - TA98,TA97a) b: antimutagenic (all concentrations + AFL) (SIM - TA98) b: antimutagenic (> 7.95 + SAZ) (SIM - TA98) b: antimutagenic (all concentrations + H₂O₂) (SIM - TA102) b: antimutagenic (> 1.99 + MMC), (SIM - TA102)	Lira et al., 2008
*Byrsonima correifolia* Leaves	MTT Neutral Red LDH Aquabluer Flow cytometry	cell lines: GAS, HepG2	Cytotoxicity Cell proliferation Cell cycle analysis	EtOH/H₂O	- 10 (0.08 - 39): Cytotoxicity - 3 (0.08 - 1.2): Cell Proliferation, Flow Cytometry	- ↓ cell viability: > 1.2 (HepG2); > 0.6 (GAS) - no effects on cell proliferation - ↓ G1 (all concentrations - HepG2) - ↑ subG1 (> 0.31 HepG2); ↓ G1 and ↑ subG1 (> 0.31 - GAS) - ↑ G2/M (1.2 - (GAS)	Specian et al., 2016
*Byrsonima correifolia* Leaves	Apoptosis/Necrosis CBMN Comet assay Fluorescence spectroscopy RT-qPCR	cell lines: MNP01, HepG2	Cytotoxicity Genotoxicity Mutagenicity Intracellular ROS Gene expression	MeOH/H₂O	- 3 (0.08 - 1.2): Cytotoxicity, genotoxicity, mutagenicity, ROS - 1 (1.2): RT-qPCR	- apoptotic: 1.2 (HepG2) - non-genotoxic (HepG2 - 3h); genotoxic 1.2 (HepG2 - 24h) - mutagenic: 1.2 (HepG2); non-mutagenic (MNP01) - non-oxidant (HepG2, MNP01) - ↓ *CYP1A1, ERCC4, BCL2* (1.2 - HepG2) - ↑ *AIFM1* (1.2 - MNP01)	Specian et al., 2023
*Byrsonima crassa* Leaves	Ames Assay	*S*. Typhimurium *(TA98, TA100, TA97a, TA102)*	Gene mutation	a. MeOH b. MeOH/H₂O c. CHCl₃ d. AqF e. EtAcF	a. 7 (1.2 - 21.2) b. 10 (0.6 - 23.5) c. 8 (0.2 - 12.0) d. 5 (0.54 - 8.82) e. 5 (0.42 - 6.81)	a: mutagenic: TA98 (-S9): > 15.9 b: non-mutagenic c: non-mutagenic d: non-mutagenic e non-mutagenic	Cardoso et al., 2006
*Byrsonima crassifolia* Leaves	Apoptosis/Necrosis differential stain CBMN Comet assay Fluorescence spectroscopy RT-qPCR	cell lines: MNP01, HepG2	Apoptosis/Necrosis Genotoxicity Antigenotoxicity Mutagenicity Antimutagenicity Intracellular ROS Gene expression	MeOH/H₂O	- 3 (0.15 - 2.4): Apoptosis/necrosis, Genotoxicity, Mutagenicity, ROS, Antigenotoxicity, Antimutagenicity - 1 (0.6): RT-qPCR	- non-apoptotic; non-genotoxic - antigenotoxic (0.6 + B[a]P - HepG2) (PRE, SIM, POST) - antimutagenic (0.6 +B[a]P - HepG2) (PRE, SIM, POST); (0.6 + DXR - MNP01) (PRE, SIM) - non-oxidant;- antioxidant (0.6 + H₂O₂ - MNP01) - ↓ *ERCC4*, XPC; ↑ *GCLC* (0.6 + B[a]P - HepG2) (PRE) - ↓ *GSR*, *XPC* (0.6 + B[a]P - HepG2) (POST) - ↑ *NFE2L2, GCLC* (0.6 + DXR - MNP01) (PRE) - ↓ *GSR, GCLC, GPX4* (0.6 + DXR - MNP01) (POST)	Specian et al., 2023
*Byrsonima fagifolia (B. crassifolia)* Leaves	MTT Neutral Red LDH Aquabluer Flow cytometry	cell lines: GAS and HepG2	Cytotoxicity Cell proliferation Cell cycle analysis	EtOH/H₂O	- 10 (0.15 - 78): Cytotoxicity - 3 (0.15 - 2.4): Cell Proliferation, Flow Cytometry	- ↓ cell viability: > 19.5 (HepG2); > 4.8 (GAS) - no effects on cell proliferation (HepG2, GAS) - cell cycle: ↑ G1, ↑ S (0.61 - HepG2); ↓ G1, ↑ S (2.4 - GAS)	Specian et al., 2016
*Byrsonima intermedia* Leaves	Ames Assay	*S*. Typhimurium *(TA98, TA100, TA97a, TA102)*	Gene mutation	a. MeOH b. MeOH/H₂O c. CHCl₃	a. 8 (0.2 - 11.7) b. 9 (0.2 - 13.5) c. 8 (0.2 - 10.8)	a: non-mutagenic b: non-mutagenic c: non-mutagenic	Sannomiya et al., 2007a
*Byrsonima intermedia* Leaves	MTT Neutral Red LDH Aquabluer Flow cytometry	cell lines: GAS, HepG2	Cytotoxicity Cell proliferation Cell cycle analysis	EtOH/H₂O	- 10 (0.15 - 78): Cytotoxicity - 3 (0.15 - 2.4): Cell Proliferation: Flow Cytometry	- ↓ cell viability: > 2.4 (HepG2, GAS) - no effects on cell proliferation (HepG2, GAS) - cell cycle: ↓ G1 (0.15, 0.61), ↑ G2/M (0.15) ↓ S (2.4) (HepG2); ↓ G1, ↑ S and G2/M (all concentrations) (GAS)	Specian et al., 2016
*Byrsonima intermedia* Leaves	Apoptosis/Necrosis differential stain CBMN Comet assay Fluorescence spectroscopy RT-qPCR	cell lines: MNP01, HepG2	Apoptosis/Necrosis Genotoxicity Antigenotoxicity Mutagenicity Antimutagenicity Intracellular ROS Gene expression	MeOH/H₂O	- 3 (0.15 - 2.4): Apoptosis/necrosis, genotoxicity, mutagenicity, ROS - 1 (0.6): Antigenotoxicity, antimutagenicity, RT-qPCR	- non-apoptotic; - non-genotoxic; - non-mutagenic - antigenotoxic (0.6 + B[a]P - HepG2) (PRE, SIM, POST) - antimutagenic: (0.6 + B[a]P - HepG2) (PRE, SIM, POST); (0.6 + DXR - MNP01) (PRE, SIM) - non-oxidant; antioxidant effects (0.6 + H₂O₂ - MNP01, HepG2) - ↓ *ERCC4,* ↑ *GCLC*, ↑*SOD1*, ↑ *XPC* (0.6 + B[a]P - HepG2) (PRE) - ↓ *GSR, GPX4, XPA, XPC;* ↑ *CYP1A1* (0.6 + B[a]P - HepG2) (POST) - ↑ *GCLC, GPX4* (0.6 + DXR - MNP01) (PRE) - ↓ *GCLC, GPX4* (0.6 + DXR - MNP01) (POST)	Specian et al., 2023
*Byrsonima verbascifolia* Leaves	MTT Neutral Red LDH Aquabluer Flow cytometry	cell lines: GAS, HepG2	Cytotoxicity Cell proliferation Cell cycle analysis	EtOH/H₂O	- 10 (0.15 - 78): Cytotoxicity - 3 (0.15 - 2.4): Cell Proliferation, Flow Cytometry	- ↓ cell viability: > 2.4 (HepG2); > 1.2 (GAS) - no effects on cell proliferation - cell cycle: ↓ G1, ↑ S, ↑ G2/M (GAS)	Specian et al., 2016
*Byrsonima verbascifolia* Leaves	Apoptosis/Necrosis CBMN Comet assay Fluorescence spectroscopy RT-qPCR	cell lines: MNP01, HepG2	Apoptosis/Necrosis Genotoxicity Mutagenicity Intracellular ROS Gene expression	MeOH/H₂O	- 3 (0.15 - 2.4): Apoptosis/necrosis, genotoxicity, mutagenicity, ROS - 1 (2.4): RT-qPCR	- apoptotic: 0.6 and 2.4 (HepG2) - non-genotoxic (HepG2 - 3h); non-oxidant - genotoxic > 0.6 (HepG2 - 24h) - mutagenic: all concentrations (HepG2) - ↓ *CYP1A1*; ↑ *BAX, TNF* (2.4 - HepG2) - ↑ *TNF* (2.4 -MNP01)	Specian et al., 2023
*Davilla elliptica* Leaves	Ames Assay	*S*. Typhimurium *(TA98, TA100, TA97a, TA102)*	Gene mutation	a. MeOH b. EtOH c. AqF d. EtAcF	a. 5 (1.0 - 12.5) b. 5 (1.0 - 12.5) c. 6 (2.6 - 61.6) d. 6 (1.3 - 31.6)	a: mutagenic: TA98 (+S9): all concentrations b: mutagenic: TA98 (+S9): 1 and 2.1 c: non-mutagenic d: non-mutagenic	Biso et al., 2010
*Davilla nitida* Leaves	Ames Assay	*S*. Typhimurium *(TA98, TA100, TA97a, TA102)*	Gene mutation	a. MeOH b. MeOH/H₂O c. AqF d. EtAcF	a. 4 (1.9 - 11.3) b. 5 (0.7 - 7.5) c. 5 (7 - 84) d. 4 (11 - 13.4)	a: mutagenic: TA98 (+S9): all concentrations; TA97a (-S9): 11.3 b: mutagenic: TA98 (+S9): 1.3 and 2.6 c: non-mutagenic d: mutagenic: TA98 (-S9 /+S9) and TA97a (+S9): all concentrations	Biso et al., 2010
*Fridericia platyphylla* Roots	MTT Neutral Red LDH Apoptosis/Necrosis Flow cytometry Fluorescence spectroscopy CBMN RT-qPCR	cell lines: GAS, ACP02, HepG2	Cytotoxicity Apoptosis Cytostasis Intracellular ROS Gene expression	EtOH/H₂O	- 11 (5 - 100): Cytotoxicity - 4 (5 - 120): Apoptosis - 3 (5 - 60): Cytostasis, CBMN, ROS, RT-qPCR	- ↓ cell viability (MTT): > 30 (ACP02, HepG2); >40 (GAS) - ↓ cell viability (Neutral Red): > 30 ( ACP02); > 40 (HepG2, GAS) - ↓ cell viability (LDH): > 40 ( HepG2); >50 (ACP02); > 90 (GAS) - necrosis: 60, 120 (GAS) - non-cytostatic (GAS, ACP02); mutagenic (NBUDs): 30 (ACP02) - ↑ subG1: (60, ACP02, GAS); (30, ACP02) - non-oxidant - GAS: ↑ *CCND1* (60)*;* ACP02: ↓*BCL-XL, BIRC5, MET, TP53* (5 and 30)	Serpeloni et al., 2020
*Indigofera suffruticosa* Aerial parts	Ames Assay	*S*. Typhimurium *(TA98, TA100, TA97a, TA102)*	Gene mutation	a. MeOH b. FEF c. AEF	a. 5 (1.25 - 7.5) b. 5 (0.12 - 2.5) c. 4 (0.12 - 1.5)	a: non-mutagenic b: non-mutagenic c: non-mutagenic	Calvo et al., 2011
*Indigofera truxillensis* Aerial parts	Ames Assay	*S*. Typhimurium *(TA98, TA100, TA97a, TA102)*	Gene mutation	a. MeOH b. GLEF c. FEF d. AEF	a. 5 (1.25 - 7.5) b. 5 (0.12 - 2.5) c. 5 (0.12 - 2.5) d. 4 (0.12 - 1.5)	a: mutagenic: TA98 (-S9): > 3.75 b: non-mutagenic c: non-mutagenic d: non-mutagenic	Calvo et al., 2011
*Machaerium hirtum* Leaves	Ames Assay	*S*. Typhimurium *(TA98, TA100, TA97a, TA102)*	Gene mutation	EtOH/H₂O	- 6 (0.8 - 16): Mutagenicity - 5 (0.25 - 4): Antimutagenicity	- non-mutagenic - antimutagenic: NPD (TA98, -S9, > 0.25); (TA100, +S9, > 0.25); AFB1(TA100, +S9, > 0.25); B[a]P (TA98, +S9, > 0.25)	Ribeiro et al., 2016
*Machaerium hirtum* Leaves	CBMN MTT Apoptosis/Necrosis RT-qPCR	HepG2 cell line	Cell viability Cell death Mutagenicity Antimutagenicity Gene expression	EtOH/H₂O	- 11 (0.6 - 625): MTT - 3 (0.6 - 9.7): CBMN, Apoptosis/Necrosis, Flow Cytometry - 1 (2.4): RT-qPCR	- cytotoxicity: > 39; - non-mutagenic, non- apoptotic - antimutagenic: (2.4 + B[a]P) - (PRE, SIM, POST) - antiapoptotic: (2.4 + (B[a]P) - (PRE, SIM, POST) - cell cycle arrest: ↑ G2/M >0.6 - (2.4 + B[a]P): ↓ *CAT* and *GPX1*	Ribeiro et al., 2016
*Miconia albicans* Aerial parts	Clonogenic CBMN	V79 cell line	Cell Survival Mutagenicity Antimutagenicity	MeOH	- 8 (5 -100): Clonogenic - 3 (5 - 20): CBMN	- cytotoxicity: ≥ 40 - non-mutagenic - antimutagenic: (> 5 + DXR) (PRE, SIM, POST)	Serpeloni et al., 2011
*Miconia cabucu* Aerial parts	Clonogenic CBMN	V79 cell line	Cell Survival Mutagenicity Antimutagenicity	MeOH	- 8 (5 - 100): Clonogenic - 3 (5 - 20): CBMN	- cytotoxicity: ≥ 40 - non-mutagenic - antimutagenic: (> 5 + DXR) (PRE, SIM, POST)	Serpeloni et al., 2011
*Miconia rubiginosa* Aerial parts	Clonogenic CBMN	V79 cell line	Cell Survival Mutagenicity Antimutagenicity	MeOH	- 8 (5 - 100): Clonogenic - 3 (5 - 20): CBMN	- cytotoxicity: ≥ 40 - non-mutagenic - antimutagenic: (> 5 + DXR) (PRE, SIM, POST)	Serpeloni et al., 2011
*Miconia stenostachya* Aerial parts	Clonogenic CBMN	V79 cell line	Cell Survival Mutagenicity Antimutagenicity	MeOH	- 8 (5 - 100): Clonogenic - 3 (5 - 20): CBMN	- cytotoxicity ≥ 60 - non-mutagenic - antimutagenic: (> 5 + DXR) (PRE, SIM, POST)	Serpeloni et al., 2011
*Mouriri elliptica* Leaves	Ames	*S*. Typhimurium *(TA98, TA100, TA97a, TA102)*	Gene mutation	a. CH₂Cl₂ b. MeOH	a. 5 (1.9 - 15.2) b. 5 (2.3 - 18.6)	a: non-mutagenic b: mutagenic: TA98 (+S9), TA97a (-S9/+S9): > 11.6	Santos et al., 2008
*Mouriri pusa* Leaves	Ames	*S*. Typhimurium *(TA98, TA100, TA97a, TA102)*	Gene mutation	a. CH₂Cl₂ b. MeOH c. TEF d. FEF	a. 5 (1.7 - 13.80) b. 5 (2.8 - 22.7) c. 6 (2.8 - 44.8) d. 6 (1.5 - 24.3)	a: non-mutagenic b: mutagenic: TA98 (+S9), TA97a (-S9/+S9): > 11.6 c: mutagenic: TA98 (-S9/+S9): > 5.6 d: mutagenic: TA98, TA100 (-S9/+S9): > 1.5	Santos et al., 2008
*Myrcia bella* Leaves	MTT Proliferation curves CBMN Flow cytometry Apoptosis/Necrosis Fluorescence spectroscopy RT-qPCR	cell lines: ACP02, GAS	Cytotoxicity Cell proliferation Mutagenicity Antimutagenicity DNA content Intracellular ROS Gene expression	EtOH/H₂O	- 11 (5 - 500): Cytotoxicity - 3 (5 - 200): Cell Proliferation, Flow Cytometry, ROS, RT-qPCR - 3 (5 - 100): CBMN - 3 (5 - 400): Apoptosis	- ↓ cell viability: > 150 (GAS) and > 100 (ACP02) - ↓ cell proliferation: 200 (GAS, ACP02) - mutagenic (NBUDs): 100 (ACP02) - antimutagenic (100 + DXR - GAS, ACP02) (PRE, POST) - ↑ subG1 (200 - GAS); ↓G2/M, S (5 - ACP02) (100, 200 - GAS, ACP02) - ↑ necrosis (200, 400; oxidant (100, 200 - ACP02) - antioxidant: (> 1, GAS, ACP02) - ↑ *CCND1* (100 + DXR - GAS, ACP02), ↓ *NFE2L2* (200 - ACP02)	Serpeloni et al., 2015
*Pouteria ramiflora* Leaves	Neutral Red LDH Apoptosis/Necrosis Flow Cytometry Immunocytochemistry RT-qPCR	cell lines: HepG2, FGH	Cytotoxicity Cell cycle kinetics Gene expression	EtOH/H₂O	- 3 (0.5 - 2): Apoptosis, Flow Cytometry, Immunocytochemistry - 1 (1): RT-qPCR	- ↑ apoptotic (HepG2 > 1.0; FGH > 0.5) - HepG2 - ↑apoptotic: 1.0 + CIS (PRE, SIM); FGH - 1.0 + CIS (SIM) - HepG2 - ↓ G1 and ↑ S, G2/M > 0.5 - HepG2 (1.0 + CIS) - ↑ S, ↓G1 (PRE, SIM, POST) - HepG2 (1.0 + CIS) - ↑ PCNA, ↓ Cyclin D1 (PRE, SIM, POST) - HepG2 - ↑ *BAX,* ↑ *CDK2* (SIM)	Tuttis et al., 2021
*Qualea grandiflora* Bark	Ames	*S.* Typhimurium *(TA98, TA100, TA97a, TA102)*	Gene mutation	a. MeOH b. CHCl₃ c. AqE	a. 5 (2.26 - 18.1) b. 5 (1.92 - 15.4) c. 5 (1.25 - 10)	a: mutagenic: TA100 (-S9): > 13.57 b: mutagenic: TA100 (-S9), TA97a (-S9/+S9), TA102 (+S9): > 11.55 c: mutagenic: TA100 (+S9), TA102 (-S9): 1.25	Santos et al., 2011
*Qualea multiflora* *Bark*	Ames	*S.* Typhimurium *(TA98, TA100, TA97a, TA102)*	Gene mutation	a. MeOH b. CHCl₃ c. AqE	a. 5 (2.5 - 20) b. 5 (2.07 - 16.6) c. 5 (1.25 - 10)	a: mutagenic: TA100 (-S9): > 10 b: mutagenic: TA100 (-S9/+S9): > 8.3 c: mutagenic: TA98 (-S9): > 3.75	Santos et al., 2011
*Qualea parviflora* *Bark*	Ames	*S.* Typhimurium *(TA98, TA100, TA97a, TA102)*	Gene mutation	MeOH	- 5 (2.84 - 22.7)	a: non-mutagenic	Mazzolin et al., 2010
*Serjania marginata* Leaves	MTT Proliferation curve CBMN Flow cytometry Fluorescence spectroscopy RT-qPCR	cell lines: ACP02, MNP01	Cytotoxicity Cell proliferation Cell cycle kinetics Mutagenicity Antimutagenicity Intracellular ROS Gene expression	a. EtOH/H₂O	- 11 (5 - 500): Cytotoxicity - 3 (5 - 200): Cell proliferation, Flow cytometry, ROS - 3 (5 - 100): CBMN, RT-qPCR	- ↓ cell viability: > 100 (ACP02); > 300 (MNP01) - ↓ cell proliferation: 200 (72h - ACP02) - ↑ SubG1 100 and 200 (MNP01, ACP02); ↓ G1 and ↑ G2/M (100 + DXR - MNP01, ACP02) - non-mutagenic - antimutagenicity: (100 + DXR - MNP01) (PRE, POST) - cytostatic: 100 (MNP01); 50, 100 (ACP02) - pro-oxidant 100, 200 (ACP02) - antioxidant: 100 (MNP01, ACP02) - MNP01: ↑ *CAT (5), GPX1 (5), NFE2L2 (5), GSR* (100 + DXR), *TP53* (10 0+ DXR)*,* and *CCND1* (100, 100 + DXR) - ACP02: ↑ *CCND1* (100 + DXR), ↓ *TP53* (50, 100), *NFE2L2* and *GSR* (100)	Serpeloni et al., 2021
*Strychnos pseudoquina* Leaves	Ames	*S*. Typhimurium *(TA98, TA100, TA97a, TA102)*	Gene mutation	a. CH₂Cl₂ b. MeOH c. AEF d. FEF e. IF	a. 5 (2.3 - 18.8) b. 5 (3.3 - 26.6) c. 5 (4.6 - 15.6) d. 5 (1.7 - 13.7) e. 4 (3.0 - 15.2)	a: non-mutagenic b: mutagenic: TA98 (-S9), TA100 (-S9/+S9): 19.9 and 26.6) c: non-mutagenic d: non-mutagenic e: non-mutagenic	Biso et al., 2010

(-S9): without metabolic activation; (+S9): with metabolic activation; AIFM1: Apoptosis-inducing factor mitochondria associated 1; ACP02: Gastric adenocarcinoma; AEF: Alkaloids Enriched Fraction; AFB1: Aflatoxin B1; AFL: Aflatoxin; AqE: Aqueous Extract; AqF: Aqueous Fraction; B[a]P: Benzo[a]pyrene; BAX: BCL2 Associated X; BCL2: B-cell lymphoma 2; BCL-XL: B-cell lymphoma-extra-large; BIRC5: Baculoviral inhibitor of apoptosis repeat containing 5; CA: Chromosome Aberration; CAT: Catalase; CBMN: Cytokinesis-Block Micronucleus Assay; CCND1: Cyclin D1; CDK2: Cyclin-dependent kinase 2; CH₂Cl₂: Dichloromethane extract; CHCl₃: Chloroform extract; CIS: Cisplatin; CYP1A1: Cytochrome P450 family 1 subfamily A member 1; DXR: Doxorubicin; ERCC4: Excision Repair Cross-Complementation Group 4; EtAcF: Ethyl Acetate Fraction; EtOH/H₂O: Hydroethanolic extract; FGH: Human gingival fibroblasts; FEF: Flavonoids Enriched Fraction; GAS: Primary normal gastric epithelium; GCLC: Glutamate-cysteine ligase catalytic subunit; GLEF: Glycerol Lipides Enriched Fraction; GPX1: Glutathione peroxidase 1; GPX4: Glutathione peroxidase 4; GSR: Glutathione-disulfide reductase; H₂O₂: Hydrogen Peroxide; HepG2: Hepatocellular carcinoma; IF: Intermediate Fraction; LDH: Lactate dehydrogenase; MeOH: Methanol extract; MeOH/H₂O: Hydromethanolic extract; MET: Mesenchymal epithelial transition receptor; MMC: Mitomycin C; MMS: Methyl Methanesulfonate; MNP01: Non-tumor gastric epithelium; MTT: 3-[4,5-dimethylthiazol-2-yl]-2,5 diphenyl tetrazolium bromide; NBUDs: Nuclear Buds; NDI: Nuclear Division Index; NFE2L2: Nuclear factor, erythroid 2 Like 2; NPD: 4-Nitro-o-phenylenediamine; PCNA: Proliferating cell nuclear antigen; PRE: Pre-treatment; POST: Post-treatment; ROS: Reactive Oxygen Species; RT-qPCR: Reverse Transcription Quantitative Polymerase Chain Reaction; SAZ: Sodium azide; SIM: Simultaneous treatment; SOD1: Superoxide dismutase 1; TEF: Tannins Enriched Fraction; TNF: Tumor necrosis factor; TP53: Tumor protein p53; V79: Chinese hamster lung fibroblast; XPA: Xeroderma Pigmentosum, Complementation Group A; XPC: Xeroderma Pigmentosum, Complementation Group C

**Table 3:** Main vegetal extracts evaluated in the Laboratory of Mutagenesis and Oncogenetics and their biological activities assessed *in vivo*.

Species/organ	Assay	Model	Parameter	Extract	Concentrations: (Number/range mg/kg b.w. (via gavage	Effects	Ref.
*Alchornea castaneifolia* Leaves	MN (erythrocytes	*Mus musculus*	Mutagenicity	MeOH	- 3 (625 - 1250	a: mutagenic (1250	Santos et al., 2010
*Alchornea glandulosa* Leaves	MN (erythrocytes	*Mus musculus*	Mutagenicity	MeOH	- 3 (625 - 1250	a: mutagenic (1250	Santos et al., 2010
*Alchornea triplinervia* Leaves	MN (erythrocytes	*Mus musculus*	Mutagenicity	MeOH	- 3 (670 - 1335	a: mutagenic (1335	Calvo et al., 2010
*Byrsonima crassa* Leaves	MN (erythrocytes	*Mus musculus*	Mutagenicity	a. MeOH b. MeOH/H₂O c. CHCl₃ d. AqF e. EtAcF	a. 3 (200 - 540 b. 3 (200 - 540 c. 3 (200 - 540 d. 3 (200 - 540 e. 3 (200 - 540	a: non-mutagenic b: non-mutagenic c: non-mutagenic d: non-mutagenic e: non-mutagenic	Cardoso et al., 2006
*Byrsonima intermedia* Leaves	MN (erythrocytes	*Mus musculus*	Mutagenicity	a. MeOH b. MeOH/H₂O c. CHCl₃	a. 3 (200 - 540 b. 3 (200 - 540 c. 2 (400 and 540	a: non-mutagenic b: mutagenic (400 and 540 c: non-mutagenic	Sannomiya et al., 2007a
*Croton cajucara* Bark	MN (bone marrow Lethal Dominant	*Mus musculus*	Mutagenicity Antimutagenicity Reproductive Toxicity	MeOH	- 3 (312.5 - 1250	- non-mutagenic - no reproductive toxicity effect - non-antimutagenic	Santos et al., 2006
*Croton cajucara* Bark	MN (erythrocytes Histological Analysis	*Mus musculus*	Mutagenicity Antimutagenicity Testicular Toxicity	MeOH	- 3 (312.5 - 1250	- non-mutagenic - antimutagenic (all concentrations + DXR - no testicular toxicity - no protective effects on testicular alterations induced by DXR	Caneguim et al., 2011
*Croton cajucara* Bark	MN (erythrocytes	*Mus musculus*	Mutagenicity	MeOH	- 3 (312.5 - 1250	- non-mutagenic - antimutagenic (all concentrations + CPA	Santos et al., 2008
*Davilla elliptica* Leaves	MN (erythrocytes	*Mus musculus*	Mutagenicity	a. MeOH/H₂O b. EtOH/H₂O	a. 3 (200 - 540 b. 3 (200 - 540	a: non-mutagenic b: non-mutagenic	Biso et al., 2010
*Davilla nitida* Leaves	MN (erythrocytes	*Mus musculus*	Mutagenicity	MeOH/H₂O	- 3 (200 - 540	- non-mutagenic	Biso et al., 2010
*Miconia albicans* Aerial parts	MN (erythrocytes Comet (erythrocytes	*Mus musculus*	Mutagenicity Antimutagenicity Genotoxicity Antigenotoxicity	a. MeOH b. CHCl₃	a. 3 (200 - 540 b. 3 (200 - 540	a,b: non-mutagenic; a,b: antimutagenic (540 + CPA a,b: genotoxic (all concentrations a,b: antigenotoxic (540 + CPA	Serpeloni et al., 2008a
*Miconia albicans* Aerial parts	MN (bone marrow	*Mus musculus*	Antimutagenicity	a. MeOH b. CHCl₃	a. 1 (540 b. 1 (540	- a,b: antimutagenic (CPA	Serpeloni et al., 2008a
*Miconia cabucu* Aerial parts	MN (erythrocytes Comet (erythrocytes	*Mus musculus*	Mutagenicity Antimutagenicity Genotoxicity Antigenotoxicity	MeOH	- 3 (200 - 540	- non-mutagenic; - antimutagenic (540 + CPA - genotoxic (all concentrations - antigenotoxic (540 + CPA	Serpeloni et al., 2008a
*Miconia cabucu* Aerial parts	MN (bone marrow	*Mus musculus*	Antimutagenicity	MeOH	- 1 (540	- non-antimutagenic (CPA	Serpeloni et al., 2008b
*Miconia rubiginosa* Aerial parts	MN (erythrocytes Comet (erythrocytes	*Mus musculus*	Mutagenicity Antimutagenicity Genotoxicity Antigenotoxicity	MeOH	- 3 (200 - 540	- non-mutagenic - antimutagenic (540 + CPA - genotoxic (all concentrations - antigenotoxic (540 + CPA	Serpeloni et al., 2008a
*Miconia rubiginosa* Aerial parts	MN (bone marrow	*Mus musculus*	Antimutagenicity	MeOH	- 1 (540	- antimutagenic (540 + CPA	Serpeloni et al., 2008b
*Miconia stenostachya* Aerial parts	MN (erythrocytes Comet (erythrocytes	*Mus musculus*		MeOH	- 3 (200 - 540	- non-mutagenic - antimutagenic (540 + CPA - genotoxic (all concentrations - antigenotoxic (540 + CPA	Serpeloni et al., 2008a
*Miconia stenostachya* Aerial parts	MN (bone marrow	*Mus musculus*	Antimutagenicity	MeOH	- 1 (540	- antimutagenic (540 + CPA	Serpeloni et al., 2008b
*Mouriri pusa* Leaves	MN (erythrocytes	*Mus musculus*	Mutagenicity	MeOH	- 3 (665.0 - 1330	- non-mutagenic	Santos et al., 2013
*Qualea grandiflora* Bark	MN (erythrocytes	*Mus musculus*	Mutagenicity	MeOH	- 3 (909.0 - 1818	- non-mutagenic	Santos et al., 2013
*Qualea multiflora* Bark	MN (erythrocytes	*Mus musculus*	Mutagenicity	MeOH	- 3 (855.8 - 1711.6	- non-mutagenic	Santos et al., 2013
*Strychnos pseudoquina* Leaves	MN (erythrocytes	*Mus musculus*	Mutagenicity	MeOH	- 3 (900 - 1800	- mutagenic (1800	Biso et al., 2010

AqF: Aqueous Fraction; CHCl₃: Chloroform extract; CPA: Cyclophosphamide; DXR: Doxorubicin; EtAcF: Ethyl Acetate Fraction; EtOH/H₂O: Hydroethanolic extract; MN: Micronucleus; MeOH: Methanol extract; MeOH/H₂O: Hydromethanolic extract

Panel (B) of Figure 2 presents the distribution of outcomes across the assays performed for the evaluated species. Twenty of the species tested exhibited at least one extract with no evidence of genotoxicity/mutagenicity. Among these, six species had extracts identified as antigenotoxic/antimutagenic in at least one assay. Interestingly, six species contain extracts or compounds classified as non-genotoxic, genotoxic, and chemopreventive. For example, extracts from species of the genus *Miconia* were characterized as non-mutagenic and antimutagenic in the CBMN assay. However, the *in vivo* comet assay and the micronucleus assay in rodent erythrocytes revealed that these extracts are genotoxic, non-mutagenic, antigenotoxic, and antimutagenic.

### Phytochemicals and biological activities

Based on the results with the standardized extracts, we sought to evaluate the biological activities, mainly antitumoral, of isolated phytochemicals. In total, 13 manuscripts assessed 8 phytochemicals in LAMON, with 7 phytochemicals evaluated *in vitro:* brachydin A (BrA), brachydin B (BrB), brachydin C (BrC), cirsimarin (CIR), trans-dehydrocrotonin (DCTN), trans-crotonin (CTN), isatin (ISA), and indirubin (IRN); and 4 *in vivo*: DCTN, CTN, IRN, and ISA. A summary of the results obtained in these studies is presented in Table 4.

**Table 4:** A summarized list of phytochemicals, their classes, sources, and biological activities evaluated *in vitro* and *in vivo* in the Laboratory of Mutagenesis and Oncogenetics.

Phytochemical/ Chemical structure	Class/ Plant/Source	Model (cell line/animal)	Parameter	Assay	Number of concentrations/ range (µM)	Effects	Ref.
***In vitro***							
**BrA** 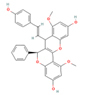	Flavonoid *Fridericia platyphylla*/roots	DU-145 (3D culture)	1. Cytotoxicity 2. Proliferation 3. Cell death 4. Migration and Invasion 5. Cell metabolism 6. Protein expression	a. Acid Phosphatase Assay b. Resazurin Assay c. Clonogenic assay d. Spheroid volume e. Annexin V-FITC f. 3D ECM Gel® g. Invasion h. High-Content Screening i. Western blotting j. RT-qPCR	a, b. 6 (10 - 100) c, e, f, g, h. 4 (40-100) d. 6 (10-100) i, j. 2 (80 and 100)	a. ⭡ cytotoxicity (48 h, ≥ 60) b. ⭡ cytotoxicity (24 h, 100) c. ↓ survival fraction (40 - 100) d. ⭡ spheroid volume (72 h, 40 - 100) e. ⭡ apoptosis (72h, > 60) f. ↓ cell migration (24 h, 60 - 100) g. ↓ invasiveness (48 h, > 60) h. ⭡ apoptosis (24 h and 48 h) and ⭡ necrosis (24 - 72 h) > 60; ↓ Δψm (0.5 h) > 60 i. ↓ MMP, ⭡ apoptosis and necrosis markers; ⭡cleaved-PARP and p-γ-H2AX - 80 and 100 j ↓ *BCL-2, BAD, RIP3K, p-AKT1, p-44/42,* and *MAPK;* ⭡ *CASP3, CASP7, CASP8, NF-kB* and *TNF-α* (80 and 100 µM)	(Ribeiro et al., 2022
**BrA** 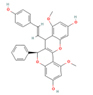	Flavonoid *Fridericia platyphylla*/roots	DU-145 PNT-2 (2D culture)	1. Cytotoxicity 2. Proliferation 3. Cell death 4. Migration and Invasion 5.Oxidant/antioxidant	a. Resazurin b. Neutral red c. MTT d.Clonogenic assay e. Proliferation curves f. Triple-stained g. LDH release h. Wound healing i. Transwell assays j. CM-H_2_DCFDA	a. 9 (1 - 60) - PNT2; 9 (0.24 - 30.62) - DU145 b, c, g, j. 9 (0.24 - 30.62) - DU145 d, e. 4 (0.24 - 15.36) - PNT2/DU145 f. 6 (1 - 60) - PNT2; 3 (1.5 - 24) - DU145 h. 3 (1.5 - 6) - DU145; 3 (1 - 5) - PNT2 i. 2 (3.84 and 6) - DU145	a. ⭡ cytotoxicity: PNT2 cells (24 h, 40) and DU-145 (24 h, 6) b, c, g. ⭡ cytotoxicity: Neutral red (IC_50_ 9.89 µM), MTT assay (IC_50_ 6.08), and LDH test (IC_50_ 12.78) d. ↓ proliferation (15.36 - DU-145) e. No antiproliferative effects (96 h - DU145) f. No induction of cell death (PNT2); ⭡ necrosis (1.5 - 24) and ⭡ apoptosis at 24 (DU-145) h. ↓ migration: PNT2 (>2.5, 48h) i. ↓ vertical migration but not invasion (24 h, 6) j. No oxidant/antioxidant	(Oliveira et al., 2021b)
**BrB** 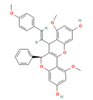	Flavonoid *Fridericia platyphylla*/roots	DU-145 (2D and 3D cultures)	1. Cytotoxicity 2. Cell death 3. Proliferation 4. Migration and Invasion 5. Oxidant/antioxidant	2D a. MTT b. LDH release c. Clonogenic assay d. Proliferation curves e. Tryple-stained f. Wound healing g. Transwell assays 3D h. Resazurin i. 3D ECM gel	2D a, b. 9 (0.24 - 30.72) c, d. 4 (0.24 - 15.36) e. 3 (1.5 - 24) f. 3 (1.5 - 6) g. 2 (3.84 and 6) 3D h, i. 7 (5 - 60)	a, b. ⭡ cytotoxicity: MTT assay (≥ 1.5), LDH assay (≥ 30.72) c. ↓ proliferation: (≥ 0.24) d. No antiproliferative effects (72 h) e. No induction of cell death (24 h) f. ↓ migration (48 h, 6) g. ↓ vertical migration and invasion (24 h, 6) h. ⭡ cytotoxicity: 24 h (IC_50_ 837.3), 48 h (IC_50_ 522.5), 72 h (IC_50_ 472.3), 168 h (IC_50_ 104.9) i. ↓ migration: 24 and 48 h (≥ 30)	(Serpeloni et al., 2023)
***BrC*** 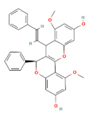	Flavonoid *Fridericia platyphylla/roots*	DU145 (2D and 3D cultures)	1. Cytotoxicity 2. Proliferation 3. Cell death 4. Oxidant/antioxidant 5. Migration/Invasion 6. Gene and protein analysis	2D a. Resazurin assay b. LDH release c. Clonogenic assay d. Proliferation curves e. CM-H_2_DCFDA f. Wound healing g. Transwell assays 3D h. Resazurin assay i. LDH release j. CM-H_2_DCFDA k. 3D ECM gel l. RT-qPCR m. Western blotting	2D a, b. 9 (0.24 - 30.72) c, d, e, 4 (0.24 - 15.36) f. 3 (1.5 - 6) g. 2 (3.84 and 6) 3D h, i, j, k 7 (5 - 60) l. 2 (5 and 50) m. 2 (50 and 60)	a, b. ⭡ cytotoxicity: Resazurin (IC_50_ 47.31), LDH assay (IC_50_ 188.7) c, d. No antiproliferative e. No oxidant/antioxidant f. ↓ migration (48 h, 6) g. ↓ vertical migration and invasion (24 h, 6) h, i. ⭡ cytotoxicity: Resazurin 24 h (IC_50_ 229.8), 48 h (IC_50_ 210.5), 72 h (IC_50_ 116.1), and 96 h (IC_50_ 65.02), LDH assay (IC_50_ 104.8) j. No oxidant/antioxidant k. ↓ migration: 24 h (≥ 50) and 48 h (≥ 5) l. ⭡ *TNF-α, CASP3, CDH1* (50), *NKX3.1* (5, 50), ↓ *MMP9* (5), *MMP11, BIRC5* (5 and 50), and *ITGAM* (50) m. ↓ proteins TNF-α (60),⭡ CASP7, and BAX (60)	(Oliveira et al., 2022)
**BrA BrB BrC**	Flavonoid *Fridericia platyphylla*/roots	PC3 (2D culture)	1. Cytotoxicity 2. Genotoxicity 3. Oxidant/antioxidant 4. Protein expression	a. MTT b. Neutral red c. LDH release d. Triple staining assay e. Comet assay f. CM-H_2_DCFDA g. Western blotting	a, b, c. 9 (0.24 -30.72) - BrA, BrB, and BrC d. 3 (1.5 - 6) - BrA, BrB, and BrC e. 3 (1.5 - 6) - BrA, BrB, and BrC f. 4 (0.96 - 6) - BrA, BrB, and BrC g. BrA (3.84 or 6); BrB and BrC (3.84)	a. ↓ cell viability > 15.36 (BrA), > 3.84 (BrB), and 3.84 (BrC) at 24 h b. ↓ cell viability > 15.36 (BrA), > 6 (BrB), and 3.84 (BrC) at 24 h c. ↓ cell viability > 15.36 (BrA), > 3.84 (BrB), and 6 (BrC) at 24 h d. ⭡ necrosis (BrA 6, 24 h), ⭡ apoptosis and necrosis (BrB 6, 24 h), ⭡ apoptosis (BrC 1.5 - 6, 24 h) e. No genotoxicity f. ⭡ ROS (BrA 6, 24 h) g. ⭡ p21 (BrB and BrC 3.84); ↓ phospho-AKT (BrA 6 and BrB 3.84); ⭡ cleaved PARP (BrA, BrB, and BrC 3.84, and BrA 6)	(Nunes et al., 2020)
**CIR** 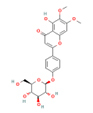	Flavonoid *Scoparia dulcis/aerial parts*	MCF-7 (2D and 3D cultures)	1. Cytotoxicity 2. Proliferation 3. Cell death 4. Oxidant/antioxidant 5. Genotoxicity 6. Migration/Invasion 7. Gene expression	2D a. Resazurin assay b. Wound healing c. Transwell assays d. Clonogenic assay 3D e. Resazurin f. Proliferation g.Triple staining h. CM-H_2_DCFDA i. Comet assay j. 3D ECM gel k. RT-qPCR	2D a, b, d. 6 (10 - 320) c. (10 - 80) 3D e, f, g, h, j. 6 (10 - 320) i, k. 2 (40 and 80)	a. ↓ cell viability (> 80, 24 h; > 40, 24 and 48 h) b. ↓ horizontal migration (> 40, 48 h) c. ↓ cell vertical invasion (10 - 80, 24 h) d. ↓ proliferation (> 10) e. ↓ cell viability (> 40, 24, 48, and 72 h) f. ↓ proliferation (> 40, day 7) g. ⭡ apoptosis (> 40, 72 h) h. No oxidant/antioxidant activity i. ⭡ DNA damage (40 and 80, 24 h) j. ↓ migration (40 and 80, 48 h) k. ↓ *CCR5, CDH1, MMP9, MMP11, CCND1, CCNA2, CDK2, CDK4, TNF, TP53, BCL-XL, BAX, CASP9*, and *BIRC5* (40 and 80, 24 h)	(Serpeloni et al., 2022)
**DCTN** 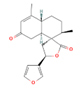	Diterpene *Croton cajucara*/barks	CHO-k1 (2D culture)	1. Proliferation 2. Cell death 3. Mutagenicity 4. Antimutagenicity	a. Nuclear division index b. Apoptosis index c. Cytokinesis-Block d. Cytokinesis-Block Micronucleus Assay	a, b, c. 5 (80 - 400) d. 2 (240 and 400) (PRE, SIM, POST)	a. No antiproliferative b. No change in the apoptosis index c. No clastogenicity d. ⭡ anticlastogenic against MMS (PRE, 240 and 400; SIM, 240; POST, 400), MMC (PRE, 240 and 400; POST, 240 and 400), and DXR (PRE, 240 and 400)	(Poersch et al., 2007)
**ISA** 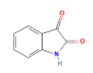	Indolinone purchased from Fluka	CHO-k1 Hela	1. Cytotoxicity 2. Cell death 3. Proliferation 4. Mutagenicity	a. MTT assay b. Apoptosis index c. Nuclear division index d. Micronucleus	a. 9 (0.1 - 1000) b, c, d. 5 (0.5 - 50)	a. ↓ cell viability (200 - 1000, 3 and 24 h) - CHO-K1 a. ↓ viability/proliferation (500 - 1000, 24 h) - HeLa b. ⭡ apoptosis (10 - 50, 24 h) - CHO-K1/HeLa cells c. ↓ proliferation (10 -50, 24 h) - CHO-K1/HeLa d. No mutagenicity on CHO-K1 and HeLa	(Cândido-Bacani et al., 2013)
**IRN/ISA** 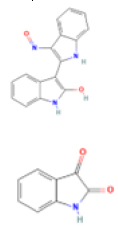	Indolinone Alkaloid purchased from Fluka	CHO-k1 Hela	1. Cytotoxicity 2. Cell death 3. Genotoxicity 4. Mutagenicity 5. Gene expression	a. MTT assay b. Trypan blue c. Acridine orange/ethidium bromide d. Cytokinesis-blocked micronucleus e. Nuclear division index f. Comet assay g. RT-qPCR	a. 10 (0.1 - 200) - CHO-k1 and HeLa - IRN b. 3 (0.2 - 5) - IRN; 3 (0.5 - 50) ISA - HeLa c, e. 5 (0.1 - 10) - IRN d. 3 (50 - 150) - IRN f. 3 (0.2 - 5) - IRN/ISA g. 1 (5, IRN; 50, ISA)	a. ↓ cell viability: CHO-K1 (5 - 200); HeLa (5 - 200) b. No cytotoxicity c. No apoptotic d. No mutagenicity e. ↓ cell proliferation (0.5 - 10.0, 24 h) - CHO-k1 and (5 - 10, 24 h) - HeLa f. 3h: ⭡ genotoxicity (IRN, 0.2 - 5) and (ISA, 0.5 - 50 ) - HeLa f. 24 h: ⭡ genotoxicity (1-5, IRN) and (IRN, 0.5 - 50, ISA) - HeLa g. No alterations in *BAX* and *ERCC1*	(Fogaça et al., 2017)
***In vivo* range (mg/kg b.w)**							
**DCTN** 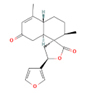	Diterpene *Croton cajucara*/barks	*Mus musculus*	1. Antimutagenicity	a. Micronucleus b. Chromosome aberration	a, b. 3 (138.75 - 416.25) intraperitoneal a, b. 3 (138.75 - 416.25) by gavage	a, b. ↓ mutagenicity (277.5 and 416.25, 24 h) against CPA intraperitoneal a, b. ↓ mutagenicity (138.75 - 416.25, 24 h) by gavage	(Agner et al., 2001)
**DCTN** 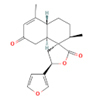	Diterpene *Croton cajucara*/barks	*Mus musculus*	a. Mutagenicity	a. Micronucleus b. Chromosome aberration	a, b. 3 (138.75 - 416.25) intraperitoneal	a, b. Non-mutagenic	(Agner et al., 1999)
**DCTN/CTN** 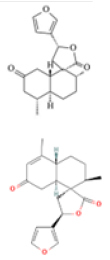	Diterpene *Croton cajucara*/barks	*Mus musculus*	a. Xenograft tumors (Sarcoma 180 and Ehrlich carcinoma ascitic tumors) b. Apoptosis	a. Sarcoma 180 and Ehrlich tumor growth b. DNA electrophoresis	a. 2 (80 and 120) intraperitoneal	a. ⭡ antitumor activity against Sarcoma 180 and Ehrlich carcinoma: DCTN (80 and 120), CTN (no activity) b. No apoptotic	(Maciel et al., 2000)
**IRN** 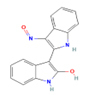	Alkaloid purchased from Fluka	*Mus musculus*	a. Genotoxicity b. Mutagenicity	a. Comet assay b. Micronucleus	a, b. 3 (50 - 150) by gavage	a. ⭡ genotoxicity (100 and 150, 4 h) b. Non-mutagenic	(Fogaça et al., 2017)
**ISA** 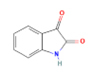	Indolinone purchased from Fluka	*Mus musculus*	a. Genotoxicity	a. Comet assay b. Micronucleus	a, b. 3 (50 - 150) by gavage	a. ⭡ genotoxicity (150) b. ⭡ mutagenicity (150)	(Cândido-Bacani et al., 2011)

BAD: BCL2 Associated Agonist Of Cell Death; CCND1: Cyclin D1; BAX: BCL2 Associated X; BCL-XL: B-cell lymphoma-extra-large; BCL2: B-cell lymphoma 2; BIRC5: Baculoviral inhibitor of apoptosis repeat containing 5; BrA: Brachydin A; BrB: Brachydin B; BrC: Brachydin C; CASP3: Caspase 3; CASP7: Caspase 7; CASP8: Caspase 8; CASP9: Caspase 9; CCNA2: Cyclin A2; CCR5: C-C Motif Chemokine Receptor 5 ; CDH1: Cadherin 1; CDK-4: Cyclin-dependent kinase 4; CDK2: Cyclin-dependent kinase 2; CHO-K1: Chinese Hamster; Ovary, Normal tissue; CIR: Cirsimarin; CPA: Cyclophosphamide; CTN: trans-crotonin; DCTN: Trans -dehydrocrotonin; DU-145: Human prostate cancer cell line derived from metastatic site: brain

DXR: Doxorubicin; ERCC1: ERCC Excision Repair 1, Endonuclease Non-Catalytic Subunit; Hela: Human; cervix; Adenocarcinoma; IRN: Indirubin; ISA: Isatin; ITGAM: Integrin Subunit Alpha M; MAPK: Mitogen-Activated Protein Kinase; MCF-7: Human Mammary Gland cell line, Breast; Derived From Metastatic Site: Pleural Effusion; MMC: Mitomycin C; MMP: Mitochondrial membrane potential; MMP11: Matrix Metallopeptidase 11; MMP9: Matrix Metallopeptidase 9; MMS: Methyl methanesulfonate; NF-kB: Nuclear Factor Kappa B Subunit ; p-AKT1: phosphorylated AKT Serine/Threonine Kinase 1; PC3: Human prostate derived From metastatic site bone; PNT2: Human prostate normal tissue immortalized with SV-40

POST: Post-treatment; PRE: Pre-treatment; RIP3K: Receptor Interacting Serine/Threonine Kinase 3; RT-qPCR: Reverse Transcription Quantitative Polymerase Chain Reaction; SIM: Simultaneous treatment; TNF-α: TNF: Tumor necrosis factor; *TP53*: Tumor protein p53.

*In vitro* assays predominantly employed prostate cancer cell lines (DU-145 and PC-3) and the breast cancer cell line MCF-7, alongside non-tumor lines PNT-2 (human prostate) and CHO-K1 (Chinese hamster ovary). Except for DCTN, all tested compounds exhibited antitumor activity, including cytotoxic, antiproliferative, and antimigratory effects. DCTN was the only isolated compound evaluated for antimutagenic potential, exhibiting protective effects against genotoxic damage induced by methyl methanesulfonate (MMS), mitomycin C (MMC), and doxorubicin (DXR) in CHO-K1 cells *in vitro*, as well as against cyclophosphamide (CPA)-induced genotoxicity *in vivo* in mice. All *in vivo* assays used Swiss albino mice.

**Figure 3:**
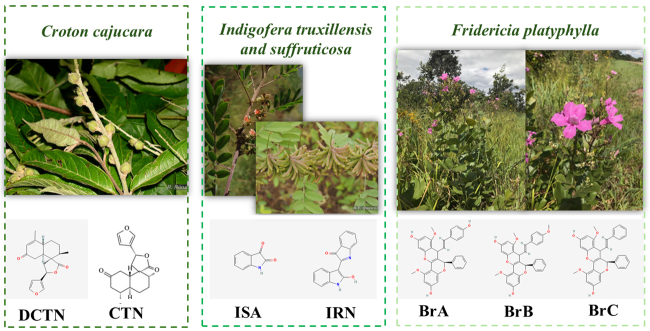
Representative images of the aerial parts of the three most evaluated plant species in the Laboratory of Mutagenesis and Oncogenetics, along with the chemical structure of the phytochemicals that can be obtained from them. Source: Plant images: https://floradobrasil.jbrj.gov.br; Chemical structures: https://pubchem.ncbi.nlm.nih.gov and (Esteves-Souza et al., 2018).

Phytochemicals derived from *C. cajucara* (DCTN and CTN), *Indigofera* (ISA and IRN), and *F. platyphylla* (BrA, BrB, and BrC) were further discussed due to their extensive evaluation in the LAMON studies (Figure 3).

## Discussion

### *Croton cajucara* and DCTN

*C. cajucara* Benth (family Euphorbiaceae), commonly known as “sacaca”, is a medicinal plant traditionally used in folk medicine, particularly in the Amazon region of Brazil (Lima et al., 2024). The stem bark is typically prepared as a herbal tea for treating various conditions, including diabetes, diarrhea, stomach pain, fever, hepatitis, and malaria (Maciel et al., 2000 *,* 2002; Lima *et al.,* 2024). Phytochemical analyses of *C. cajucara* extracts identified DCTN, a 19-nor-clerodane diterpene, as the primary bioactive compound in the bark, linked to most of its reported pharmacological activities (Maciel *et al.,* 2000). Among its demonstrated biological effects are antiulcerogenic (Rodríguez et al., 2004), antitumor (Lapenda et al., 2013), and hypoglycemic properties (Morais et al., 2009). Furthermore, DCTN has shown antioxidant, antigenotoxic, and cardioprotective effects, expanding its pharmacological profile (Agner et al., 2001; Silva et al., 2005b). 

Given the promising potential of compounds derived from these Amazonian species and the need for comprehensive evaluation of their biological effects and toxicity profiles, LAMON initiated genotoxicity and antigenotoxicity studies in the late 1990s, examining both the *C. cajucara* plant extract and the isolated compound DCTN.

The mutagenicity of DCTN was assessed in mouse bone marrow cells using the micronucleus test and chromosomal aberration analysis (Agner et al., 1999). DCTN was solubilized in dimethyl sulfoxide (DMSO) and administered intraperitoneally at doses of 138.7, 277.5, and 416.2 mg/kg b.w.. In this study, treatment with the diterpene obtained from sacaca did not affect the mitotic index or significantly alter the frequency of micronuclei or cells with chromosomal aberrations in treated animals compared to the negative control group.

Complementarily, the antimutagenic activity of DCTN against CPA-induced *in vivo* effects was evaluated using the same assays (Agner et al., 2001). The results demonstrated that DCTN, at doses of 277.5 and 416.2 mg/kg b.w., exhibited statistically significant antimutagenic effects, reducing both the frequency of micronuclei and the number of cells with chromosomal aberrations in animals co-treated with CPA.

Poersch et al. (2007) evaluated the *in vitro* clastogenic, antigenotoxic, and cytotoxic effects of DCTN in CHO-K1 cells exposed to MMS, MMC, and DXR. DCTN (80-400 µM) showed no clastogenic or apoptotic activity but exhibited significant antigenotoxic effects, reducing micronucleus frequencies by up to 61% (pre-treatment with 400 µM DCTN against MMC). Protection varied by mutagen and protocol: pre-treatment was most effective (e.g., 48.5% reduction against MMS), whereas simultaneous and post-treatment showed selective efficacy (e.g., 25.7% reduction with 240 µM DCTN + MMC). DCTN also attenuated apoptosis induced by all mutagens, particularly MMS (31.3-34.8% apoptosis vs. 48.0% in control). Cytotoxicity was negligible, though higher DCTN concentrations (320-400 µM) increased necrosis. The study confirmed DCTN’s desmutagenic and bioantimutagenic potential, with mechanisms likely involving the modulation of reactive oxygen species (ROS) and direct DNA protection.

A multidisciplinary investigation assessed crude extracts, DCTN, and two additional predominant terpenoids from *C. cajucara* bark: AAA (acetyl aleuritolic acid) and CTN (Maciel et al., 2000). The study reinforced the therapeutic potential of these compounds, with DCTN emerging as the most promising candidate based on its antitumor properties.

Specifically, the clerodane diterpenoid DCTN demonstrated significant *in vivo* antitumor activity against both Sarcoma 180 and Ehrlich carcinoma ascitic models in DBA/2 mice. At doses of 84-120 mg/kg, DCTN increased survival time by 128-140% (T/C ratio), demonstrating efficacy comparable to that of 5-fluorouracil (5-FU). Complementary *in vitro* studies revealed potent cytotoxicity against Ehrlich carcinoma cells (IC₅₀ = 16 µM for both DCTN and CTN) (Maciel et al., 2000). Even though mechanistic studies indicated that Tumor Necrosis Factor-α human (TNF-α) mediated immunomodulatory effects (Grynberg et al., 1999), none of the compounds induced tumor cell apoptosis; these collective findings highlight DCTN’s potential as a lead antitumor agent.

Complementing prior evidence on the safety *of C. cajucara* derivatives, Santos et al. (2006) conducted a subchronic (28-day) evaluation of methanol stem-bark extracts from sacaca (312.5-1250 mg/kg) in Swiss mice using bone marrow micronucleus and dominant lethal assays. The study confirmed the absence of mutagenicity in both somatic and germ cells, with PCE frequencies (1.1-1.4/1000 cells) indistinguishable from those of the negative control (1.2/1000 cells). Additionally, testosterone levels remained unaffected, further supporting the safety of the extract for reproductive tissues. However, the extract failed to mitigate CPA-induced DNA damage (150 mg/kg), suggesting limited chemoprotective efficacy in hematopoietic stem cells. These findings align with the established low toxicity of DCTN (Agner et al., 2001).

Expanding on these results, Santos et al. (2008) demonstrated that methanol stem-bark extracts (MeOH - 312.5-1250 mg/kg) exhibited dose-dependent antimutagenicity against CPA-induced DNA damage in Swiss mice, as assessed by micronucleus assays in MNRET. While no intrinsic mutagenicity was observed (MNRET frequencies: 0.9-1.6/1000 cells, comparable to negative control), the two highest doses (625 and 1250 mg/kg) significantly reduced CPA-induced micronuclei from the first week of treatment (P < 0.05), suggesting rapid desmutagenic activity. This contrasts with the lower dose (312.5 mg/kg), which required three weeks to show efficacy, implying a cumulative effect. These findings align with the absence of genotoxicity reported by Santos *et al.* (2006) in bone marrow and germ cells, but expand on this by revealing the extract’s protective role against the alkylating agent CPA.

The *in vivo* antimutagenicity and reproductive toxic effects of the MeOH extract from the stem bark of *C. cajucara* (312.5-1250 mg/kg) were also evaluated by Caneguim et al. (2011) in mice exposed to DXR. The study revealed that, while the extract demonstrated no intrinsic mutagenicity, it exhibited significant antigenotoxic activity by reducing DXR-induced DNA damage, as evidenced by a decrease in MNRET frequencies (up to a 79% reduction at 625 mg/kg). However, MeOH failed to mitigate DXR-induced testicular toxicity, including seminiferous tubule atrophy, germ cell depletion, and fertility impairment, suggesting tissue-specific limitations in cytoprotection. 

The Euphorbiaceae family already contains a phytochemical, the diterpene ester derived from *Euphorbia peplus*, known as Ingenol mebutate, which is approved for topical use in the chemopreventive treatment of precancerous and cancerous skin lesions (Shafombabi et al., 2025). Complementarily, recent nanotechnological advances, particularly controlled-release systems like SNEDDS (self-nanoemulsifying drug delivery systems), have been explored to enhance DCTN bioavailability and therapeutic efficacy, offering new perspectives for pharmaceutical applications (Lima et al., 2024). Oliveira Netto et al. (2025) developed an SNEDDS using copaiba oil resin as a carrier for t-DCTN. They described it as a potential antioxidant nanoproduct warranting further investigation of its biological activities. 

Collective evidence demonstrates that *C. cajucara* and its primary diterpenoid, DCTN, exhibit a safe genotoxic profile and consistent antigenotoxic properties across *in vitro* and *in vivo* models, with mechanisms involving ROS modulation and direct DNA protection. While extracts and DCTN show no intrinsic mutagenicity, their chemoprotective efficacy varies by tissue and the nature of the genotoxic damage, underscoring the need for further research to optimize therapeutic applications while addressing tissue-specific limitations.

### *Indigofera*, isatin, and indirubin

The genus *Indigofera* belongs to the tribe Indigofereae in the Fabaceae family with over 750 species distributed in most tropical to temperate areas of the world (Du Preez et al., 2025); however, only 3 species were found in Brazil: *Indigofera suffruticosa* Miller*, Indigofera truxillensis* H. B. Kunt*,* and *Indigofera hirsuta* Lindl*.*, all considered weeds. In LAMON, different extracts from two species found in Brazil, *I. suffruticosa* and *I. truxillensis*, were evaluated.

The MeOH extracts from aerial parts of both species, as well as the glycerolipid, flavonoid, and alkaloid fractions, were evaluated for their mutagenic effects using TA100, TA98, TA102, and TA97a strains in the Ames test. *I. truxillensis* was mutagenic in the TA98 strain without S9, while the glycerolipid fraction was not. *I. suffruticosa* had no mutagenic activity, but it had mutagenic index values around 2 (TA98-S9), suggesting the presence of potentially mutagenic compounds. The flavonoid and alkaloid fractions of both plants exhibited mutagenic properties (Calvo et al., 2011)**.**

Other studies in the literature have also reported significant biological activities for these extracts. Recently, Tran et al. (2024) showed that the extract from *I. suffruticosa* aerial parts exhibited cytotoxic effects in various leukemic cell lines, induced G2/M cell cycle arrest, promoted apoptosis via increased caspase-3/7 activity, and enhanced DNA damage, as evidenced by elevated phospho-H2A.X levels. These species were also evaluated in *in vivo* studies. Lima et al. (2019) assessed the MEOH extract of *I. suffruticosa* leaves on Swiss albino mice and demonstrated hepatoprotective activity and high antioxidant potential. For *I. truxillensis,* Luiz-Ferreira et al. (2012) assessed the aqueous and ethyl acetate extracts obtained from leaves; they showed *in vivo* that the extracts acted as gastroprotective agents, stimulating protective factors and antioxidant enzymes.

Two isolated phytochemicals from *I. suffruticosa* and *I. truxillensis*, ISA and IRN, were also evaluated at LAMON. These compounds were initially isolated from the plant extracts and later purchased from Fluka due to insufficient quantities for complete evaluation. First, Cândido-Bacani et al. (2011) evaluated *in vivo* the genotoxic (comet assay) and mutagenic (micronucleus test) effects of acute (24h) and repeated (14d) exposure to ISA in different doses (50, 100, and 150 mg/kg b.w.) The LD_50_ to mice was estimated in 1 g/kg b.w. and at all doses ISA induced genotoxicity but not mutagenicity in acute treatments. After repeated doses, only ISA (150 mg/kg b.w.) induced MNPCE and MNRET in mice, highlighting the importance of evaluating subchronic and chronic protocols for DNA damage. Although ISA induced DNA damage, no cytotoxicity was observed when the PCE/NCE ratio was calculated. 

After detection of genotoxic and mutagenic effects *in vivo,* Cândido-Bacani et al. (2013) evaluated the possible mechanisms of action of ISA *in vitro* using CHO-K1 and HeLa cells. ISA was cytotoxic to CHO-K1 and HeLa cells at concentrations ≥ 200 µM and ≥500 µM, respectively. At a concentration of 50 µM, ISA was not mutagenic in either cell line. Fogaça et al. (2017) reported no cytotoxic effects after treating HeLa cells with 50 µM ISA in the trypan blue assay. However, it inhibited cell proliferation (as indicated by the PCE/NCE ratio) and promoted apoptosis *in vivo*. The anticancer activity of ISA was also evaluated by another research group, which demonstrated its ability to inhibit neuroblastoma cell proliferation, invasion, and migration *in vitro*, as well as to prevent distant metastasis in tumor-bearing mice (Hua et al., 2021).

Another active compound IRN, obtained from *Indigofera* extracts, was evaluated at LAMON both *in vitro* and *in vivo*. *In vitro*, IRN was more cytotoxic than ISA, reducing the viability of CHO-K1 (≥5 μM) and HeLa cells (10 μM) at lower concentrations (Fogaça et al. 2017). IRN also presented antiproliferative effects (nuclear division index, NDI) in both cell lines (≥0.5 and ≥5.0 to CHO-K1 and HeLa cells, respectively) and genotoxicity in the comet assay. However, it did not induce apoptosis, micronuclei, or altered *expression of BAX* (BCL2 Associated X, Apoptosis Regulator) and *ERCC1* (ERCC Excision Repair 1, Endonuclease Non-Catalytic Subunit) *in vitro*. *In vivo*, IRN (100 and 150 mg/kg) also induced genotoxicity. A recent publication, using multiple bioinformatic tools including immunoinformatic and AI algorithms, demonstrated that IRN is a promising drug candidate and could be further explored as a colon-rectal cancer inhibitor with minimal side effects (Ali et al., 2025). More interestingly, Yeo et al. (2025) developed an optimized bioconversion process to produce a high yield of indirubin from recycled waste jeans, thereby improving the compound’s production for screening and large-scale applications.

### *Fridericia platyphylla* and brachydins

The genus *Fridericia* comprises approximately 60 species, distributed across a range of ecosystems, including moist and dry forests, as well as Cerrado and Caatinga vegetation, extending from Mexico to Argentina and southern Brazil (Henrique et al., 2024). *F. platyphylla* is a member of the Bignoniaceae family, native to the Brazilian Cerrado. Brazilians consumed their roots to treat kidney stones and joint pains (Monteiro et al., 2020). Crude extracts of this species were investigated in the Biota/FAPESP Project. The main biological activities of the extract include antiulcer (Rocha et al., 2017), antileishmanial (Rocha et al., 2014), anti-inflammatory (Salgado et al., 2020), antinociceptive (Da Rocha et al., 2011), antimicrobial (Sousa et al., 2020), and mutagenic (Resende et al., 2017). 

The antitumor activity of the crude hydroethanolic extract from the roots of *F. platyphylla* was evaluated in LAMON using *in vitro* models of gastric and liver cancer. Serpeloni et al. (2020) assessed the selective cytotoxicity of the extract using the MTT assay (3-[4,5-dimethylthiazol-2-yl]-2,5 2,5-diphenyl tetrazolium bromide) on non-tumor (GAS) and tumor-derived (ACP02 and HepG2) cells. The extract showed low selectivity, with IC_50_ values of 56.16, 43.68, and 42.57 µg/mL for GAS, ACP02, and HepG2 cells, respectively. The extract induced cell death, arrested the cell cycle, and modulated genes related to both pathways, including *BCL-XL* (BCL2-like 1)*, BIRC5* (Baculoviral IAP Repeat-Containing 5, also known as survivin), and *MET* (MET Proto-Oncogene, Receptor Tyrosine Kinase).

Considering the various biological activities described for the extract, a class of new compounds, known as brachydins, was isolated from the nonpolar fraction of an aqueous ethanol extract of the roots of the *F. platyphylla* by Rocha et al. (2014). Ten brachydins have already been successfully isolated (brachydins A, B, C, D, E, F, G, H, I, and J) (Rocha *et al.,* 2017), and some have been evaluated for their biological activities. In total, 11 studies assessed the biological activities of brachydins, isolated or combined, including antileishmanial (Rocha *et al.,* 2014; Costa et al., 2024; Neves et al., 2024), antitumoral (Lima et al., 2022), and anti-inflammatory (Salgado et al., 2020). The most evaluated activity is antileishmanial and has been reported to exhibit selective cytotoxicity against promastigotes of *L. infantum*, *L. braziliensis*, and *L. mexicana*, with low toxicity in RAW264.7 macrophages. For *L. amazonensis* promastigotes, the encapsulated form of BrA was also selective towards macrophages. These results demonstrate reduced toxicity of BrA compared to current reference treatments. 

Among the 11 studies described, 5 were conducted at LAMON and evaluated the *in vitro* antitumor activity of brachydins A, B, and C. Nunes et al. (2020) evaluated BrA, BrB, and BrC in 2D cultures of PC-3 prostate tumor cells. They found IC_50_ values of 23.41, 4.28, and 4.44 µM in MTT assays for BrA, BrB, and BrC, respectively; these results were confirmed in neutral red and LDH (lactate dehydrogenase) release assays. They explored these results using Western blot analysis and showed that the low IC_50_ values may be supported by increased p21 and cleaved PARP (poly(ADP-ribose) polymerase), as well as decreased pAKT (AKT serine/threonine kinase) protein levels. Additional computational comparisons using the public database PubChem from the National Center for Biotechnology Information (NCBI) showed that, among 1330 molecules with similarity to brachydins, 44 exhibited cytotoxicity in several tumor cell lines: HepG2 (liver), MCF-7 and T-47D (mammary epithelium), A549 (lungs), and SV480 (colon).

BrA was evaluated in prostate tumor DU145 and non-tumor prostate epithelial PNT-2 cells by Oliveira et al. (2021b) for its selective antitumor activity. The Selectivity Index (SI) for BrA was estimated at 7.3 (sevenfold) against the tumor DU145 cells, indicating a good selective candidate for an antitumor drug. This selectivity was confirmed in the other cell viability assays (resazurin, neutral red, MTT, and LDH release). Furthermore, BrA impaired DU145 vertical migration in transwell assays, suggesting a possible antimetastatic activity.

Both studies described (Nunes et al., 2020; Oliveira et al., 2022) were conducted using a 2D culture model (monolayer). A better success rate in translational research depends on experimental models that mimic physiological conditions in tumors, such as 3D culture models. In recent years, LAMON has screened compounds for antitumor activity using spheroids generated by forced floating or liquid overlay. In this technique, cell suspensions are seeded onto agarose-coated wells, and spheroids are then generated by centrifugation (Friedrich et al., 2009). 

The antitumorigenic effects of BrA were evaluated *in vitro* using prostate spheroids (DU145) by Ribeiro et al. (2022), in collaboration with LAMON. This brachydin was cytotoxic to DU145 tumor spheroids at concentrations ≥60 µM after 48 h of exposure; values ten times higher than those reported by Nunes et al. (2020) and Oliveira et al. (2022) using cultured 2D monolayers. Ribeiro *et al*. (2022) proposed that parthanatos cell death mediates the antiproliferative, cytotoxic, and antimetastatic properties observed in DU145 spheroids triggered by mitochondrial dysfunction and DNA fragmentation by PARP overactivation, as previously observed in 2D by Nunes *et al.* (2020). Oliveira *et al.* (2022) showed that brachydin C induced cell death and inhibited epithelial-mesenchymal transition in DU145 spheroids, which remained more resistant than 2D cultures; in this study, the authors associated death with *BIRC5* (Baculoviral IAP Repeat Containing 5, survivin) downregulation and increased expression of *TNF-α,* caspase 3, caspase 7, and *NKX3.1* (NK3 Homeobox 1), while enhanced *CDH1* (Cadherin 1) and reduced metalloproteinases *9* and *11* expression impaired cell migration. BrB presented an IC_50_ of 7.45 μM in 2D and 837.3 μM in 3D, both after 24 h of treatment. Additionally, the 3D model showed a decrease in the area and volume of 3D DU145 spheroids, as well as antimigratory effects (Serpeloni et al., 2023).

The higher concentrations needed for 3 brachydins to induce antitumor effects in spheroids are associated with their 3D architecture, proliferative gradients, hypoxic core, intercellular interactions, biomarker expression, and limited treatment penetration (Alwahsh et al., 2024). Even so, brachydins exhibited significant antitumor activity in 3D cultures, which provide a more physiologically relevant model of *in vivo* tumor behavior. Ongoing studies are now evaluating the *in vivo* antitumor effects of brachydins using a xenograft tumor model in mice.

## Final Considerations

**Figure 4:**
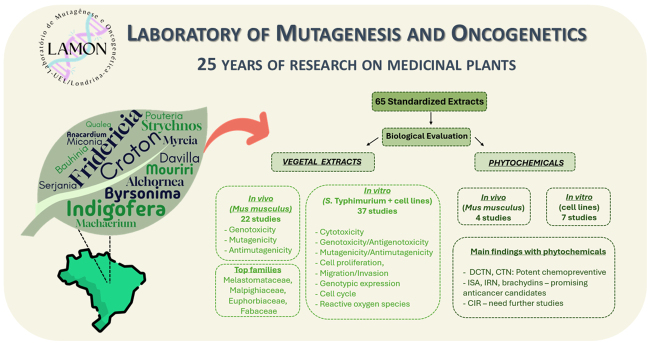
Schematic representation of the main results obtained over 25 years of research by the Laboratory of Mutagenesis and Oncogenetics (LAMON) with medicinal plants. In the image, the main genera evaluated are highlighted in the leaf, along with the results, separated into those obtained with plant extracts and those obtained with isolated active principles.

This review summarizes the promising cytotoxic, mutagenic, genotoxic, and protective activities of medicinal plant extracts commonly used by local communities in Brazil, and some of their isolated phytochemicals (Figure 4).

Most of the extracts evaluated did not exhibit harmful effects on genetic material; however, extracts from some species showed cytotoxic and mutagenic activities, indicating caution regarding their safety for use by the population. However, their anticancer properties, such as cytotoxicity and genotoxicity (among others), encouraged us to select five phytochemicals from these extracts to evaluate some of their biological activities. ISA and IRN found in the *Indigofera* genus, and brachydins (BrA, BrB, and BrC) derived from *F. platyphylla,* stood out as promising candidates for anticancer agents. On the other hand, the protective activity against genetic material exhibited by DCTN, derived from *C. cajucara*, not only indicates a promising safety profile for its widespread use but also suggests strong potential as a chemopreventive agent. 

This review provides an overview of the current scenario of the activities described for the extracts and phytochemicals studied at LAMON. However, it is essential to further investigate the studies to date, as the mechanisms of action of the six phytochemicals considered promising remain poorly understood. We hope this review of these natural products will be helpful to other groups designing new drugs based on medicinal plants.

## Data Availability

All data presented in this review are presented in the published manuscripts.

## References

[B1] Abranches S (2020). Biological megadiversity as a tool of soft power and development
for Brazil. Bras Political Sci Rev.

[B2] Agner AR, Maciel MA, Pinto AC, Cólus IM (2001). Antigenotoxicity of trans-dehydrocrotonin, a clerodane diterpene
from Croton cajucara. Planta Med.

[B3] Agner AR, Maciel MA, Pinto AC, Pamplona SG, Cólus IM (1999). Investigation of genotoxic activity of trans-dehydrocrotonin, a
clerodane diterpene from Croton cajucara. Teratog Carcinog Mutagen.

[B4] Akhtar MF, Saleem A, Rasul A, Faran Ashraf Baig MM, Bin-Jumah M, Abdel Daim MM (2020). Anticancer natural medicines: An overview of cell signaling and
other targets of anticancer phytochemicals. Eur J Pharmacol.

[B5] Albuquerque UP, Monteiro JM, Ramos MA, Amorim ELC (2007). Medicinal and magic plants from a public market in northeastern
Brazil. J Ethnopharmacol.

[B6] Ali N, Akbar R, Saleem A, Ali A, Ali A (2025). AI based natural inhibitor targeting RPS20 for colorectal cancer
treatment using integrated computational approaches. Sci Rep.

[B7] Alvarez F, Marimon-Junior BH, Marimon BS, Ter Steege H, Phillips OL, Dias Françoso Brandão R, Matricardi EA, Pinto JR, Guimarães Guilherme FA, Leandro Bueno M (2025). Tree species hyperdominance and rarity in the South American
Cerrado. Commun Biol.

[B8] Alwahsh M, Al-Doridee A, Jasim S, Awwad O, Hergenröder R, Hamadneh L (2024). Cytotoxic and molecular differences of anticancer agents on 2D
and 3D cell culture. Mol Biol Rep.

[B9] Azqueta A, Collins AR (2013). The essential comet assay: A comprehensive guide to measuring DNA
damage and repair. Arch Toxicol.

[B10] Barcelos GRM, Shimabukuro F, Maciel MAM, Cólus IMS (2007). Genotoxicity and antigenotoxicity of cashew (Anacardium
occidentale L.) in V79 cells. Toxicol in Vitro.

[B11] Barcelos GRM, Shimabukuro F, Mori MP, Maciel MAM, Cólus IMS (2007). Evaluation of mutagenicity and antimutagenicity of cashew stem
bark methanolic extract in vitro. J Ethnopharmacol.

[B12] Bieski IGC, Rios Santos F, Oliveira RM, Espinosa MM, Macedo M, Albuquerque UP, Oliveira Martins DT (2012). Ethnopharmacology of medicinal plants of the Pantanal region
(Mato Grosso, Brazil). Evid Based Complement Alternat Med.

[B13] Biso FI, Rodrigues CM, Rinaldo D, Reis MB, Bernardi CC, Mattos JCP, Caldeira-de-Araújo A, Vilegas W, Cólus IMS, Varanda EA (2010). Assessment of DNA damage induced by extracts, fractions and
isolated compounds of Davilla nitida and Davilla elliptica
(Dilleniaceae). Mutat Res Genet Toxicol Environ Mutagen.

[B14] Bonacorsi C, Fonseca LM, Raddi MSG, Kitagawa RR, Vilegas W (2013). Comparison of Brazilian plants used to treat gastritis on the
oxidative burst of Helicobacter pylori-stimulated neutrophil. Evid Based Complement Alternat Med.

[B15] Bourdy G, Chāvez MLR, Roca-Coulthard A (2004). Pharmacopoeia in a shamanistic society: the Izoceño-Guaraní
(Bolivian Chaco). J Ethnopharmacol.

[B16] Calvo TR, Cardoso CRP, Silva MAC, Santos LC, Colus IMS, Vilegas W, Varanda EA (2011). Mutagenic activity of Indigofera truxillensis and I. suffruticosa
aerial parts. Evid Based Complement Alternat Med.

[B17] Calvo TR, Demarco D, Santos FV, Moraes HP, Bauab TM, Varanda EA, Cólus IMS, Vilegas W (2010). Phenolic compounds in leaves of Alchornea triplinervia:
Anatomical localization, mutagenicity, and antibacterial
activity. Nat Prod Commun.

[B18] Calvo TR, Lima ZP, Silva JS, Ballesteros KVR, Pellizzon CH, Hiruma-Lima CA, Tamashiro J, Brito ARMS, Takahira RK, Vilegas W (2007). Constituents and antiulcer effect of Alchornea glandulosa:
Activation of cell proliferation in gastric mucosa during the healing
process. Biol Pharm Bull.

[B19] Cândido-Bacani PM, Reis MB, Serpeloni JM, Calvo TR, Vilegas W, Varanda EA, Cólus IMS (2011). Mutagenicity and genotoxicity of isatin in mammalian cells in
vivo. Mutat Res.

[B20] Cândido-Bacani PM, Mori MP, Calvo TR, Vilegas W, Varanda EA, Cólus IMS (2013). In vitro assessment of the cytotoxic, apoptotic, and mutagenic
potentials of isatin. J Toxicol Environ Health A.

[B21] Caneguim BH, Serpeloni JM, Maciel MAM, Colus IMS, Mesquita SFP (2011). Croton cajucara methanolic extract reduced the DNA-damage but did
not protect against the testicular alterations induced by
doxorubicin. J Med Plant Res.

[B22] Cardoso CRP, Cólus IMS, Bernardi CC, Sannomiya M, Vilegas W, Varanda EA (2006). Mutagenic activity promoted by amentoflavone and methanolic
extract of Byrsonima crassa Niedenzu. Toxicology.

[B23] Collia M, Møller P, Langie SAS, Vettorazzi A, Azqueta A (2024). Further development of CometChip technology to measure DNA damage
in vitro and in vivo: Comparison with the 2 gels/slide format of the
standard and enzyme-modified comet assay. Toxicology.

[B24] Corrêa MP (1926). Dicionário das plantas úteis do Brasil e das exóticas
cultivadas.

[B25] Corrêa MP (1984). Dicionário das plantas úteis do Brasil e das exóticas
cultivadas.

[B26] Corrêa MP (1978). Dicionário das plantas úteis do Brasil e das exóticas
cultivadas.

[B27] Corrêa MP (1978). Dicionário das plantas úteis do Brasil e das exóticas
cultivadas.

[B28] Costa AR, Silva JRL, Oliveira TJS, Silva TG, Pereira PS, Borba EFO, Brito ES, Ribeiro PRV, Almeida-Bezerra JW, C JT (2020). Phytochemical profile of Anacardium occidentale L. (cashew tree)
and the cytotoxic and toxicological evaluation of its bark and leaf
extracts. S Afr J Bot.

[B29] Costa CS, Marques EM, Nascimento JR, Lima VAS, Santos-Oliveira R, Figueredo AS, Jesus CM, Souza NGC, Brandão CM, Jesus ET (2024). Design of liquid formulation based on F127-loaded natural dimeric
flavonoids as a new perspective treatment for leishmaniasis. Pharmaceutics.

[B30] Du Preez B, Schrire BD, Dreyer LL, Stirton CH, Chimphango SBM, Muasya AM (2025). Global biogeographic patterns of the genus Indigofera (Fabaceae:
Indigofereae). Braz J Bot.

[B31] Duke JA, Vasquez R (1994). Amazonian ethnobotanical dictionary.

[B32] Durigan G, Pilon NAL, Assis GB, Souza FM, Baitello JB (2018). Plantas pequenas do cerrado: biodiversidade negligenciada.

[B33] Esteves-Souza A, Pissinate K, Maciel MAM, Echevarria A (2018). Synthesis of new trans-dehydrocrotonin nitrogenated derivatives
and their cytotoxic and DNA-topoisomerase I inhibitory
activities. J Braz Chem Soc.

[B34] Fenech M (2000). The *in vitro* micronucleus
technique. Mutat Res.

[B35] Fogaça MV, Cândido-Bacani PM, Benicio LM, Zapata LM, Cardoso PF, Oliveira MT, Calvo TR, Varanda EA, Vilegas W, Cólus IMS (2017). Effects of indirubin and isatin on cell viability, mutagenicity,
genotoxicity and BAX/ERCC1 gene expression. Pharm Biol.

[B36] Friedrich J, Seidel C, Ebner R, Kunz-Schughart LA (2009). Spheroid-based drug screen: Considerations and practical
approach. Nat Protoc.

[B37] Grynberg NF, Echevarria A, Lima JE, Pamplona SS, Pinto AC, Maciel MA (1999). Anti-tumour activity of two 19-nor-clerodane diterpenes,
trans-dehydrocrotonin and trans-crotonin, from Croton
cajucara. Planta Med.

[B38] Guimarães APA, Guimarães AC, Alcântara DÁ, Cunha LR, Lima PL, Vasconcellos MC, Montenegro RC, Soares BM, Amorim MM, Burbano RR, Sierra LM, Gaivão I (2014). Genotoxicity and DNA repair: A practical approach.

[B39] Henrique CY, Alves OJA, Silva MLA, Cunha WR, Januario AH, Pauletti PM (2024). O Gênero Fridericia (Bignoniaceae): Composição química e
potencial biológico. Quim Nova.

[B40] Heredia-Vieira SC, Simonet AM, Vilegas W, Macías FA (2015). Unusual C,O-fused glycosylapigenins from Serjania marginata
leaves. J Nat Prod.

[B41] Hiruma-Lima CA, Calvo TR, Rodrigues CM, Andrade FDP, Vilegas W, Brito ARMS (2006). Antiulcerogenic activity of Alchornea castaneaefolia: Effects on
somatostatin, gastrin and prostaglandin. J Ethnopharmacol.

[B42] Hua Y, Zhou N, Zhang J, Zhang Z, Li N, Wang J, Zheng W, Li X, Wang F, Zhang L (2021). Isatin inhibits the invasion and metastasis of SH-SY5Y
neuroblastoma cells in vitro and in vivo. Int J Oncol.

[B43] Ignoato MC, Fabrão RM, Schuquel ITA, Botelho MFP, Bannwart G, Pomini AM, Arruda LLM, Bersani-Amado CA, Santin SMO (2013). Chemical constituents of Machaerium hirtum Vell. (Fabaceae)
leaves and branches and its anti-inflammatory activity
evaluation. Nat Prod Res.

[B44] Lapenda TLS, Morais WA, Almeida FJF, Ferraz MS, Lira MCB, Santos NPS, Maciel MAM, Santos-Magalhães NS (2013). Encapsulation of trans-dehydrocrotonin in liposomes: An
enhancement of the antitumor activity. J Biomed Nanotechnol.

[B45] Lima CA, Cubero MCZ, Franco YEM, Rodrigues CDP, Nascimento JR, Vendramini-Costa DB, Sciani JM, Rocha CQ, Longato GB (2022). Antiproliferative activity of two unusual dimeric flavonoids,
brachydin E and brachydin F, isolated from Fridericia platyphylla (Cham.)
L.G.Lohmann: In vitro and molecular docking evaluation. Biomed Res Int.

[B46] Lima IR, Silva IB, Lima RML, Silva TMS, Maia MBS, Leite SP (2019). Hepatoprotective efficacy of methanolic extract of Indigofera
Suffruticosa (Mill) on paracetamol-induced liver damage in
mice. Arq Gastroenterol.

[B47] Lima LR, da Silva FL, Arcanjo DDR, Medeiros Maciel MA (2024). Croton cajucara: Patents and nanotechnological
advances. Recent Pat Nanotechnol.

[B48] Lira WM, Santos FV, Sannomiya M, Rodrigues CM, Vilegas W, Varanda EA (2008). Modulatory effect of Byrsonima basiloba extracts on the
mutagenicity of certain direct and indirect-acting mutagens in Salmonella
typhimurium assays. J Med Food.

[B49] Lorenzi H (1992). Árvores brasileiras: manual de identificação e cultivo de plantas
arbóreas nativas do Brasil.

[B50] Lorenzi H (1998). Árvores Brasileiras: Manual de Identificação e Cultivo de Plantas
Arbóreas Nativas do Brasil.

[B51] Luiz-Ferreira A, Cola M, Barbastefano V, Faria FM, Almeida ABA, Farias-Silva E, Calvo TR, Hiruma-Lima CA, Vilegas W, Souza-Brito ARM (2012). Healing, antioxidant and cytoprotective properties of Indigofera
truxillensis in different models of gastric ulcer in rats. Int J Mol Sci.

[B52] Maciel MA, Pinto AC, Arruda AC, Pamplona SG, Vanderlinde FA, Lapa AJ, Echevarria A, Grynberg NF, Côlus IMS, Farias RA (2000). Ethnopharmacology, phytochemistry and pharmacology: A successful
combination in the study of Croton cajucara. J Ethnopharmacol.

[B53] Maciel MAM, Pinto AC, Jr. VF Veiga, Grynberg NF, Echevarria A (2002). Plantas medicinais: a necessidade de estudos
multidisciplinares. Quím Nova.

[B54] Marena GD, Girotto L, Saldanha LL, Ramos MADS, Grandis RA, Silva PB, Dokkedal AL, Chorilli M, Bauab TM, Pavan FR (2022). Hydroalcoholic extract of Myrcia bella loaded into a
microemulsion system: A study of antifungal and mutagenic
potential. Planta Med.

[B55] Mazzolin LP, Nasser ALM, Moraes TM, Santos RC, Nishijima CM, Santos FV, Varanda EA, Bauab TM, da Rocha LRM, Di Stasi LC (2010). Qualea parviflora Mart.: An integrative study to validate the
gastroprotective, antidiarrheal, antihemorragic and mutagenic
action. J Ethnopharmacol.

[B56] Monteiro FS, Costa JRS, Martins LJ, Rocha CQ, Borges AC, Borges MOR (2020). Hydroalcoholic extract of leaves of Arrabidaea brachypoda (DC.)
Bureau present antispasmodic activity mediated through calcium influx
blockage. Rev Ciênc Farm Básica Apl.

[B57] Morais WA, Costa MP, Paixão ADO, Maciel MAM, Santos-Magalhães NS (2009). Encapsulation and release characteristics of DCTN/PLGA
microspheres. J Microencapsul.

[B58] Neves MA, Nascimento JR, Maciel-Silva VL, Santos AM, V JJG, Coelho AJS, Lima MIS, Pereira SRF, Rocha CQ (2024). Anti-leishmania activity and molecular docking of unusual
flavonoids-rich fraction from Arrabidaea brachypoda
(Bignoniaceae. Mol Biochem Parasitol.

[B59] Newman DJ, Cragg GM (2020). Natural products sources of new drugs over the nearly four
decades from 01/1981 to 09/2019. J Nat Prod.

[B60] Nunes HL, Tuttis K, Serpeloni JM, Nascimento JR, Rocha CQ, Silva VAO, Lengert AH, Reis RM, Cólus IMS (2020). Characterization of the in vitro cytotoxic effects of brachydins
isolated from Fridericia platyphylla in a prostate cancer cell
line. J Toxicol Environ Health A.

[B61] OECD (2016). Test No. 474: Mammalian Erythrocyte Micronucleus Test, OECD Guidelines
for the Testing of Chemicals.

[B62] OECD (2023). Test No. 487: *In Vitro* Mammalian Cell Micronucleus
Test, OECD Guidelines for the Testing of Chemicals.

[B63] Oliveira CR, Cal VDLJ, Vaz DF, Fialho PB, Curiele RS, Figueira FS, Silva JRM, Falcão LT, Vieira RP (2021). Known and unknown medicinal plants used in respiratory disorders
in Brazilian folk medicine: A brief review. Braz J Nat Sci.

[B64] Oliveira LCB, Nunes HL, Ribeiro DL, Nascimento JR, Rocha CQ, Cólus IMS, Serpeloni JM (2021). Aglycone flavonoid brachydin A shows selective cytotoxicity and
antitumoral activity in human metastatic prostate (DU145) cancer
cells. Cytotechnology.

[B65] Oliveira LCB, Ribeiro DL, Nascimento JR, Rocha CQ, Cólus IMS, Serpeloni JM (2022). Anticancer activities of Brachydin C in human prostate tumor
cells (DU145) grown in 2D and 3D models: Stimulation of cell death and
downregulation of metalloproteinases in spheroids. Chem Biol Drug Des.

[B66] Oliveira JR, Corrêa NP, Aragão ALB, Paiva WS, Oliveira RHA, Morais LWA, Oliveira NJH, Santos MDC, Santos-Magalhães NS, Veiga VF (2025). Bioavailability for the improved therapeutic profile of
trans-dehydrocrotonin incorporated into a copaiba oil self-nanoemulsifying
drug delivery system: Formulation, physicochemical characterizations, and
antioxidant in vitro effect. Int J Mol Sci.

[B67] Panizza S (1997). Plantas Que Curam. Cheiro De Mato.

[B68] Poersch A, Santos FV, Maciel MAM, Câmara JKP, Castro DTN, Cólus IMS (2007). Protective effect of DCTN (trans-dehydrocrotonin) against
induction of micronuclei and apoptosis by different mutagenic agents in
vitro. Mutat Res.

[B69] Pott A, Pott VJ (1994). Plantas do Pantanal.

[B70] Pott A, Pott VJ, Souza TW (2006). Plantas daninhas de pastagem na região dos cerrados.

[B71] Resende FA, Nogueira CH, Espanha LG, Boldrin PK, Oliveira-Höhne AP, Santoro CM, Quintino RC, Vilegas W, Varanda EA (2017). In vitro toxicological assessment of Arrabidaea brachypoda (DC.)
Bureau: Mutagenicity and estrogenicity studies. Regul Toxicol Pharmacol.

[B72] Ribeiro DL, Cilião HL, Specian AFL, Serpeloni JM, Oliveira MT, Varanda EA, Vilegas W, Saldanha LL, Martínez-López W, Dokkedal AL (2018). Phytochemical study and evaluation of cytotoxicity, mutagenicity,
cell cycle kinetics and gene expression of Bauhinia holophylla (Bong.)
Steud. in HepG2 cells in vitro. Cytotechnology.

[B73] Ribeiro DL, Cilião HL, Specian AFL, Serpeloni JM, Souza MF, Tangerina MMP, Vilegas W, Boldrin PK, Resende FA, Varanda EA (2016). Chemical and biological characterisation of Machaerium hirtum
(Vell.) Stellfeld: Absence of cytotoxicity and mutagenicity and possible
chemopreventive potential. Mutagenesis.

[B74] Ribeiro DL, Tuttis K, Oliveira LCB, Serpeloni JM, Gomes INF, Lengert AH, Rocha CQ, Reis RM, Cólus IMS, Antunes LMG (2022). The antitumoral/antimetastatic action of the flavonoid brachydin
A in metastatic prostate tumor spheroids in vitro Is Mediated by
(Parthanatos) PARP-Related Cell Death. Pharmaceutics.

[B75] Rocha CQ, Vilela FC, Santa-Cecilia FV, Cavalcante GP, Vilegas W, Giusti-Paiva A, Santos MH (2015). Oleanane-type triterpenoid: An anti-inflammatory compound of the
roots Arrabidaea brachypoda. Rev Bras Farmacogn.

[B76] Rocha CQ, Faria FM, Marcourt L, Ebrahimi SN, Kitano BT, Ghilardi AF, Luiz Ferreira A, Almeida ACA, Dunder RJ, Souza-Brito ARM (2017). Gastroprotective effects of hydroethanolic root extract of
Arrabidaea brachypoda: Evidences of cytoprotection and isolation of unusual
glycosylated polyphenols. Phytochemistry.

[B77] Rocha CQ, Queiroz EF, Meira CS, Moreira DRM, Soares MBP, Marcourt L, Vilegas W, Wolfender J-L (2014). Dimeric flavonoids from Arrabidaea brachypoda and assessment of
their anti-Trypanosoma cruzi activity. J Nat Prod.

[B78] Rocha CQ, Vilela FC, Cavalcante GP, Santa-Cecília FV, Santos SL, Santos MH, Giusti PA (2011). Anti-inflammatory and antinociceptive effects of Arrabidaea
brachypoda (DC.) Bureau roots. J Ethnopharmacol.

[B79] Rodrigues J, Rinaldo D, Santos LC, Vilegas W (2007). An unusual C6-C6” linked flavonoid from Miconia cabucu
(Melastomataceae). Phytochemistry.

[B80] Rodrigues V, Carvalho DAD (2001). Levantamento etnobotânico de plantas medicinais no domínio do
Cerrado na região do Alto Rio Grande. Rev Bras Plantas Med.

[B81] Rodríguez JA, Hiruma-Lima CA, Souza BARM (2004). Antiulcer activity and subacute toxicity of trans-dehydrocrotonin
from Croton cajucara. Hum Exp Toxicol.

[B82] Saldanha LL, Vilegas W, Dokkedal AL (2013). Characterization of flavonoids and phenolic acids in Myrcia bella
Cambess. Using FIA-ESI-IT-MSn and HPLC-PAD-ESI-IT-MS Combined with
NMR. Molecules.

[B83] Salgado C, Morin H, Coriolano AN, Neff L, Quintino RC, Vilegas W, Marcourt L, Wolfender JL, Jordan O, Ferreira QE (2020). In vitro anti-inflammatory activity in arthritic synoviocytes of
A. brachypoda root extracts and its unusual dimeric
flavonoids. Molecules.

[B84] Sannomiya M, Cardoso CRP, Figueiredo ME, Rodrigues CM, Santos LC, Santos FV, Serpeloni JM, Cólus IMS, Vilegas W, Varanda EA (2007). Mutagenic evaluation and chemical investigation of Byrsonima
intermedia A. Juss. leaf extracts. J Ethnopharmacol.

[B85] Sannomiya M, Santos LC, Carbone V, Napolitano A, Piacente S, Pizza C, Souza-Brito ARM, Vilegas W (2007). Liquid chromatography/electrospray ionization tandem mass
spectrometry profiling of compounds from the infusion of Byrsonima fagifolia
Niedenzu. Rapid Commun Mass Spectrom.

[B86] Santos FV, Calvo TR, Cólus IMS, Vilegas W, Varanda EA (2010). Mutagenicity of two species of the genus Alchornea measured by
Salmonella microsome assay and micronucleus test. Rev Bras Farmacogn.

[B87] Santos FV, Andreo M, Nasser ALM, Moreira LM, Vilegas W, Cólus IMS, Varanda E (2013). Absence of mutagenicity of plants used to treat gastrointestinal
disorders. Arch Biol Sci.

[B88] Santos FV, Mesquita SFP, Faria MJSS, Poersh A, Maciel MAM, Pinto AC, Morimoto HK, Cólus IMS (2006). Absence of mutagenicity in somatic and germ cells of mice
submitted to subchronic treatment with an extract of Croton cajucara Benth.
(Euphorbiaceae). Genet Mol Biol.

[B89] Santos FV, Nasser ALM, Biso FI, Moreira LM, Santos VJSV, Vilegas W, Varanda EA (2011). Genotoxicity of polar and apolar extracts obtained from Qualea
multiflora and Qualea grandiflora. J Ethnopharmacol.

[B90] Santos FV, Silva VSVJ, Farias MJ, Mesquita SFP, Maciel MA, Pinto AC, Cólus IMS (2008). Mutagenicity and antimutagenicity of Croton
cajucara. Biologia.

[B91] Santos FV, Tubaldini FR, Cólus IMS, Andréo MA, Bauab TM, Leite CQF, Vilegas W, Varanda EA (2008). Mutagenicity of Mouriri pusa Gardner and Mouriri elliptica
Martius. Food and Chem Toxicol.

[B92] Serpeloni JM, Bisarro RM, Rodrigues J, Campaner SL, Vilegas W, Varanda EA, Dokkedal AL, Cólus IMS (2008). In vivo assessment of DNA damage and protective effects of
extracts from Miconia species using the comet assay and micronucleus
test. Mutagenesis.

[B93] Serpeloni JM, Oliveira LCB, Fujiike A, Tuttis K, Ribeiro DL, Camara MBP, Rocha CQ, Cólus IMS (2022). Flavone cirsimarin impairs cell proliferation, migration, and
invasion in MCF-7 cells grown in 2D and 3D models. Toxicol in Vitro.

[B94] Serpeloni JM, Ribeiro DL, Weiss GF, Oliveira LCB, Fujiike AY, Nunes HL, Rocha CQ, Guembarovski RL, Cólus IMS (2023). Flavonoid brachydin B decreases viability, proliferation, and
migration in human metastatic prostate (DU145) cells grown in 2D and 3D
culture models. Toxicol Res (Camb).

[B95] Serpeloni J, Mazzaron BGR, Prates MM, Yanagui K, Vilegas W, Aparecida VE, Cólus IMS (2011). Cytotoxic and mutagenic evaluation of extracts from plant species
of the Miconia genus and their influence on doxorubicin-induced
mutagenicity: An in vitro analysis. Exp Toxicol Pathol.

[B96] Serpeloni JM, Specian A, Ribeiro DL, Benício LM, Nunes HL, Franchi LP, Rocha CQ, Vilegas W, Varanda EA, Cólus IMS (2020). Fridericia platyphylla (Cham.) L.G. Lohmann root extract exerts
cytotoxic and antiproliferative effects on gastric tumor cells and
downregulates BCL-XL, BIRC5, and MET genes. Hum Exp Toxicol.

[B97] Serpeloni JM, Specian AFL, Ribeiro DL, Tuttis K, Heredia-Vieira SC, Vilegas W, Martínez-López W, Varanda EA, Cólus IMS (2021). Selective anticancer effects of Serjania marginata Casar. extract
in gastric cells are mediated by antioxidant response. Environ Toxicol.

[B98] Serpeloni JM, Specian AFL, Ribeiro DL, Tuttis K, Vilegas W, Martínez-López W, Dokkedal AL, Saldanha LL, Cólus IMS, Varanda EA (2015). Antimutagenicity and induction of antioxidant defense by
flavonoid rich extract of Myrcia bella Cambess. in normal and tumor gastric
cells. J Ethnopharmacol.

[B99] Serpeloni JM, Vilegas W, Varanda EA, Cólus IMS (2008). Avaliação in vivo da anticlastogenicidade de extratos de plantas
medicinais do gênero Miconia através do teste do micronúcleo. Semin Ciênc Biol Saúde.

[B100] Silva MA, Rafacho BPM, Hiruma-Lima CA, Rocha LRM, Santos LC, Sannomiya M, Souza-Brito ARM, Vilegas W (2005). Evaluation of Strychnos pseudoquina ST. HIL. leaves extract on
gastrointestinal activity in mice. Chem Pharm Bull.

[B101] Shafombabi NF, Knott M, Kapewangolo P, Sharifi-Rad J, Calina D (2025). Ingenol mebutate in cancer therapy: Mechanisms, clinical
applications and future directions. Med Oncol.

[B102] Silva RM, Oliveira FA, Cunha KMA, Maia JL, Maciel MAM, Pinto AC, Nascimento NRF, Santos FA, Rao VSN (2005). Cardiovascular effects of trans-dehydrocrotonin, a diterpene from
Croton cajucara in rats. Vascul Pharmacol.

[B103] Sousa ALM, Oliveira AB, Leal AL, Alcantara OFA, Portela AL, D J, Siqueira JP, Kaatz GW, Rocha CQ, Barreto HM (2020). Antimicrobial activity and inhibition of the NorA efflux pump of
Staphylococcus aureus by extract and isolated compounds from Arrabidaea
brachypoda. Microb Pathog.

[B104] Sousa FDCDD, Araújo MP, Lemos JR (2015). Ethnobotanical study with native species in a rural village in
Piauí state, Northeast Brazil. J Plant Scien.

[B105] Specian AFL, Serpeloni JM, Tuttis K, Ribeiro DL, Cilião HL, Varanda EA, Sannomiya M, Martinez-Lopez W, Vilegas W, Cólus IMS (2016). LDH, proliferation curves and cell cycle analysis are the most
suitable assays to identify and characterize new phytotherapeutic
compounds. Cytotechnology.

[B106] Specian AFL, Tuttis K, Serpeloni JM, Ribeiro DL, Nunes HL, Tangerina MMP, Sannomiya M, Varanda EA, Vilegas W, Cólus IMS (2023). Chemical characterization of Brazilian savannah Byrsonima species
(muricis) and their impact on genomic instability and chemopreventive
effects. Mutat Res Genet Toxicol Environ Mutagen.

[B107] The Brazil Flora Group (2022). Brazilian Flora 2020: Leveraging the power of a collaborative
scientific network. Taxon.

[B108] Tran H-L, Lai K-H, Chang H-S, Chen Y-S, Wang H-C, Yang S-S, Chang H-W, Hsu C-M, Yen C-H, Hsiao H-H (2024). Indigofera suffruticosa aerial parts extract induce G2/M arrest
and ATR/CHK1 pathway in Jurkat cells. BMC Complement Med Ther.

[B109] Tuttis K, Costa DLMG, Serpeloni JM, Santos LCD, Varanda EA, Vilegas W, Martínez-López W, Cólus IMS (2021). Phytochemical profile, and antiproliferative and proapoptotic
effects of Pouteria ramiflora (Mart.) Radlk. leaf extract, and its synergism
with cisplatin in HepG2 cells. J Med Food.

[B110] Witt KL, Benthem J, Kobets T, Chen G, Kelber O, Krzykwa J, MacGregor JT, Mei N, Mitchell CA, Rietjens I (2025). A proposed screening strategy for evaluating the genotoxicity
potential of botanicals and botanical extracts. Food and Chem Toxicol.

[B111] Yeo C-S, Yuk Y, Jang J-H, Pagolu R, Choi K-Y (2025). Conversion of recycled indigo from waste blue jeans into
indirubin anticancer drug. Chemosphere.

[B112] Zeiger E (2019). The test that changed the world: The Ames test and the regulation
of chemicals. Mutat Res Genet Toxicol Environ Mutagen.

[B113] Zeiger E (2023). Determination of a positive response in the Ames Salmonella
mutagenicity assay. Environ Mol Mutagen.

